# Structural Basis of High-Precision Protein Ligation and Its Application

**DOI:** 10.1021/jacs.4c10689

**Published:** 2025-01-02

**Authors:** Kelvin Han Chung Chong, Lichao Liu, Rae Chua, Yoke Tin Chai, Zhuojian Lu, Renming Liu, Eddie Yong Jun Tan, Jinxi Dong, Yek How Khoh, Jianqing Lin, Franklin L. Zhong, Julien Lescar, Peng Zheng, Bin Wu

**Affiliations:** † School of Biological Sciences, Nanyang Technological University, 60 Nanyang Drive, Singapore 636921, Singapore; ‡ NTU Institute of Structural Biology, Nanyang Technological University, EMB 06-01, 59 Nanyang Drive, Singapore 636921, Singapore; § State Key Laboratory of Coordination Chemistry, Chemistry and Biomedicine Innovation Center (ChemBIC), School of Chemistry and Chemical Engineering, Nanjing University, Nanjing 210023, PR China; ∥ Lee Kong Chian School of Medicine, Nanyang Technological University, Singapore 308232, Singapore; ⊥ Skin Research Institute of Singapore (SRIS), Singapore 308232, Singapore

## Abstract

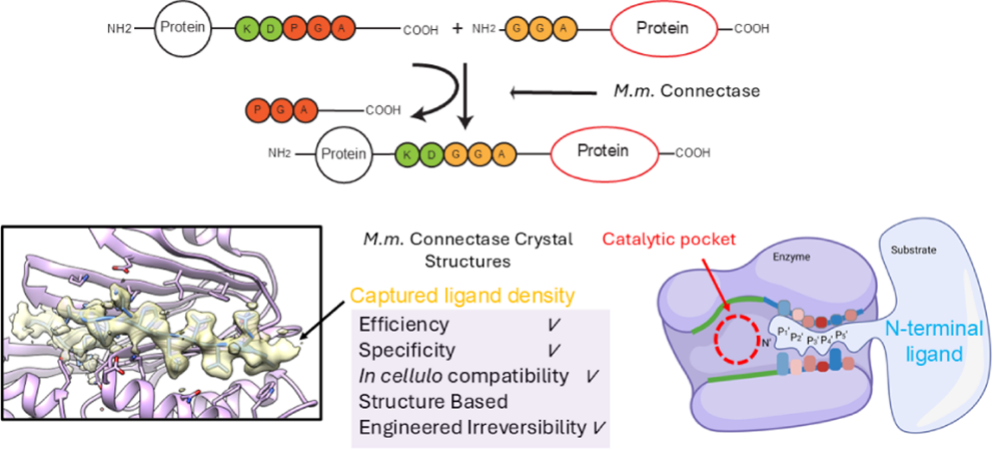

Enzyme-catalyzed protein modifications have become invaluable in diverse applications, outperforming chemical methods in terms of precision, conjugation efficiency, and biological compatibility. Despite significant advances in ligases, such as sortase A and OaAEP1, their use in heterogeneous biological environments remains constrained by limited target sequence specificity. In 2021, Lupas’ group introduced Connectase, a family of repurposed archaeal proteases for protein ligations, but its low processivity and lack of structural information have impeded further engineering for practical biological and biophysical applications. Here, we present the X-ray crystallographic structures of MmConnectase (*Methanococcus maripaludis*, MmCET) in both apo and substrate-bound forms. Comparative analysis with its inactive paralogue, MjCET (*Methanococcus janaschi*), reveals the structural basis of MmCET’s high-precision ligation activity. We propose modifications to the N-terminal substrate recognition motifs to suppress MmCET’s reversible protease activity, enabling high-precision protein ligations in complex biological environments, such as serum-containing cell cultures. To further demonstrate the enhanced processivity and precision, single-molecule protein unfolding experiments showed that our optimized Connectase, in conjunction with OaAEP1(C247A), can perform stepwise tandem ligations of protein leading to a well-defined protein polymer.

## Introduction

Since the 1990s, biochemists have endeavored to develop enzymes capable of bio-orthogonal protein and peptide modifications. Subtiligase^1,2^ and sortase A,^3^ both bacterial in origin, were repurposed early on to facilitate peptide ligation, significantly advancing protein engineering and biochemical research.^4,5^ More recently, research teams characterized plant-based asparaginyl endopeptidases (AEPs), demonstrating their potential as efficient protein ligases.^6^ In 2017, the first structure of the AEP-like protein ligase, OaAEP1, was resolved, giving unique insight into the mechanism of the transpeptidation reaction, and a hyperactive single variant, OaAEP1(C247A), was engineered by elucidating its catalytic mechanism,^7^ leading to numerous biotechnological applications.^8−10^ Despite these advances, current enzymatic ligation strategies still face limitations that hinder broader biomedical application,^11^ particularly regarding the lack of engineering potential of substrate specificity of plant AEPs, as well as the processivity of sortase A, which require further enhancement for direct in vivo use.^12−14^

The discovery of peptide ligase activity in the Connectase family of enzymes (CETs) from archaea has expanded protease repurposing strategies.^15^ However, original CETs exhibit significant residual protease activity, leading to nonprocessive (reversible) ligation and limiting their application. The substrate recognition grooves of Connectase enzymes appear amenable to engineering, with the possibility of introducing diverse substrate specificities and converting partial ligase activity into a versatile enzyme that can be used for various biotechnological applications. To date, only one inactive analog MjCET, a phylogenetic sister of Connectase, has been structurally characterized (Figure S1). Hence, a detailed structural understanding is needed to elucidate the origin of protease versus ligase activities of CETs to aid the design of more active enzymes.

In this study, we present crystal structures of a highly active protein ligase from *Methanococcus maripaludis* (MmCET) in both its apo- and substrate-bound forms. By dissecting the critical differences leading to protease versus ligase activities, we engineered a highly precise and irreversible protein ligase, capable of conducting protein ligation under near in vivo conditions. Lastly, the excellent protein ligation property of MmCET is further verified and utilized for protein unfolding experiments using atomic force microscopy-based single-molecule force spectroscopy (AFM-SMFS).^16^ AFM-SMFS is a unique tool that allows for the mechanical manipulation of single protein molecules, facilitating the study of protein (un)folding processes and measurement of protein–protein interactions.^17−21^ Recently, protein ligases such as sortase and OaAEP1(C247A) have been used for site-specific protein immobilization and polyprotein construction for AFM-SMFS studies.^16,22,23^ However, the availability of suitable ligases is currently limited.^24^ Here, we demonstrate that connectase can serve as an excellent choice for this purpose. We achieved challenging protein polymerization with controlled sequences using a combination of MmCET and OaAEP1(C247A).^16^ Thus, the connectase we obtained showed excellent protein ligation properties and has potential for applications.

## Results and Discussion

### Overall Structure of MmCNT and Comparison with Mj Connectase and Sortase

Understanding the structural features that distinguish ligase and protease activities is crucial for advancing enzymatic protein ligation techniques. To this end, we first attempted to capture MmCET in both its apo form and its peptide substrate-bound form.

Despite repeated cocrystallization attempts, holoenzyme crystals were obtained only when using the native peptide substrate. The MmCET substrate’s free form (PDB code: 8JTU) adopts a conformation reminiscent of a jaw, featuring a long groove with a plethora of side chain lining. In this conformation, cysteines in MmCET form a stable intermolecular disulfide bond (C65–C193), locking the enzyme in a dimeric state (Figure S1D) and constraining the crystal lattice. Interestingly, removing these cysteines via site-directed mutagenesis did not affect the crystallization, and ligand-free enzymes were still observed in the same crystal form. More detailed structural analysis revealed that the native substrate recognition motif is not the “optimal” combination of amino acids for MmCET recognition. Moreover, volume analysis of the substrate pocket of the free MmCET structure revealed significant potential for designing more favorable peptide substrates.

**Figure 1:**
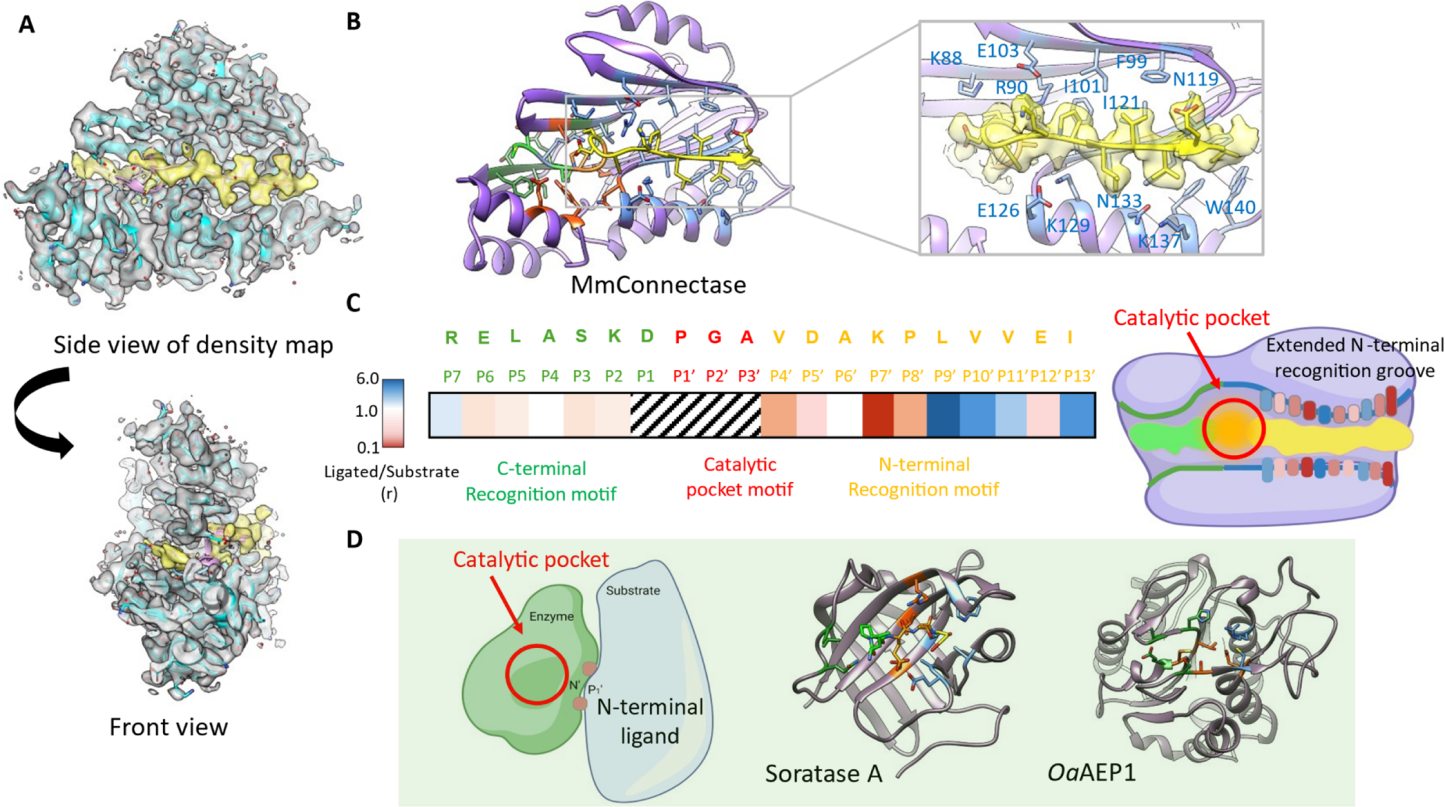
Overall X-ray crystal structure of mmConnectase (T1A) in complex with one of the enhanced recognition motifs. (A) Side view and front view of the complex electron density (transparent light gray) as the assigned atomic models (connectase in cyan and substrate peptide in yellow). (B) The side view of the atomic model with a zoom-in illustration of the N-terminal substrate (highlighted in yellow) recognition groove (enzyme residues shown in light blue). (C) The amino acid sequence of the enhanced substrate that was cocrystallized. C-terminal recognition motif (green), catalytic pocket motif (red), and N-terminal recognition motif (yellow). The color map shows the effect of alanine substitution in the indicated position of the substrate recognition motif. The measurement was determined by the ratio of the ligated product against substrates (blue: high values; white: no change; red: low values) where low values indicate unfavorable substitution for alanine in the relevant position (Figure S2). Shaded regions indicate substitutions that were eliminated from the analysis due to key positions for ligase recognition. The right panel showed a cartoon illustration of mmConnectase encompassing an extended complex substrate recognition groove. (D) Cartoon illustrations and structural views of sortase A and OaAEP1 highlighting their substrate bindng pockets.

Next, a series of peptides were synthesized and evaluated using a peptide binding and ligation assay, with mass spectrometry monitoring of peptide ligation efficacy. Swapping two amino acids at the P4’ and P7’ positions (F → V, and D → K, respectively) of MmCET (bold) significantly enhanced the ligation efficacy (peptide sequence: RELASKD|PGA**V**DA**K**PLVVEI). Subsequently, we successfully obtained a crystal structure capture of the binary complex capturing MmCET(T1A) in its catalytic intermediate state (PDB code: 8WKD). The resulting structure at ∼2.1 Å resolution revealed the atomic details of how MmCET formed intricate interactions with its preferred substrates. In contrast to the original MjCET structure reported where the bound peptide was only loosely interacting with the enzyme, here, the entire peptide was embedded in the substrate-recognizing groove (Figure 1A, electron density displayed in yellow color; Figure S1E, comparison of MjCET and MmCET binary complex structures), with the peptide sequences at the P7–P1 positions (RELASKD) accommodated in the C-terminal substrate pocket, the P1′–P3′ positions (PGA) inside the catalytic pocket (Figure S2), and the P4′–P13” positions (VDAKPLVVEI) fitting perfectly in the N-terminal substrate binding groove. Given the overall elongated shape, its recognition by MmCET can be likened to the jaw of a crocodile biting a buffalo leg. In addition, we conducted a series of mutagenesis experiments to demonstrate that the observed complex structure is indeed a snapshot of a catalytic intermediate state, of which the residues that were observed having intricate interactions with the substrate indeed have correlated impact on MmCET’s protein ligase activity (Figure S3).

### The Substrate Binding Grooves

Notably, the C-terminal substrate pocket of MmCET exhibits relatively loose interactions with the bound peptide. Sequential alanine scanning of the substrate (replacing in turn each canonical recognition sequence amino acids with alanine) at the P3–P6 positions resulted in minor changes in catalytic efficiency (Figures 1C and S3), as measured by mass spectrometry detection of the product/substrate ratio. It agreed with our observation that the sequences of peptide/protein substrates at the P7–P2 positions are relatively loosely recognized. In contrast, the N-terminal substrate recognition groove features opposite side chains that form specific interactions with the substrate. Side chains from residues 88–103, as well as N119, I121, and H123, form the upper surface of this groove, while side chains from α-helix 3 (residues 125–140) constitute the bottom surface. The bound peptide substrate within this N-terminal binding groove adopts a β-sheet secondary structure, interacting with these upper and lower surface side chains.

Among these substrate residues, the upward-facing amino acids (P7′, P8′, P10′, and P12’) interact with the upper residues. For instance, F99 and I121 from MmCET interact with the residue at the P10’ position, which prefers small-sized hydrophobic residues. I101 and F123 interact with the substrate’s P8’ residue, which is compatible with either proline or another midsized hydrophobic residue. E103 from MmCET interacts with the P7’ residue of the substrate, where lysine is preferable, explaining why an alanine replacement at P7’ is highly unfavorable. Similarly, the downward-facing residues of the substrate interact with the bottom surface. MmCET W140 and K137 interact with the substrate’s P11’ residue, which does not accommodate bulky features at this position. N133 from MmCET is in close proximity to the P9’ position of the substrate, where smaller residues are preferred. Closer to the catalytic pocket, the substrate’s P4’ and P5′ residues interact with K88 from MmCET, which accommodates amino acids with midsized or negatively charged side chains at these locations. Our alanine scan experiment agrees with these observations (Figures S4–S7).

Overall, the N-terminal substrate recognition groove (reading the P4′–P13’ residues from the substrate) forms a set of highly specific interactions, resembling the shapes of “teeth” inside the crocodile jaw, that only permitted binding of substrates with a well-defined combination of amino acids (Figure 1B,C). This observation contrasts with previously known protein ligation enzymes (Figure 1D). In sortase A, the P2′–P5′ of the substrate interacts loosely with 2–3 surface residues (highlighted in blue in Figure 1D, middle panel), allowing any N-terminal sequence with two small amino acids (Gly-Gly at P1’ and P2’) to be recognized to some extent, with no specificity for the positions from P3′ onward. This permissive recognition allows for repeated recognition of the ligation products as substrates, resulting in a highly reversible reaction. For OaAEP1 and other plant AEP-derived ligases, the P1′–P5′ substrate recognition pocket is largely lacking, leading to less restricted substrate recognition. In comparison, the extensive N-terminal substrate recognition groove (“teeth” position) in the MmCET scaffold holds significant potential for specificity engineering.

### The Catalytic Pocket

Through the analysis of the MmCET–substrate complex structure (this work), we learned about the structural origin of substrate specificity. To better understand the catalytic mechanism and distinguish a protein ligase from a protease scaffold, we conducted an in-depth analysis of the catalytic pocket.

**Figure 2:**
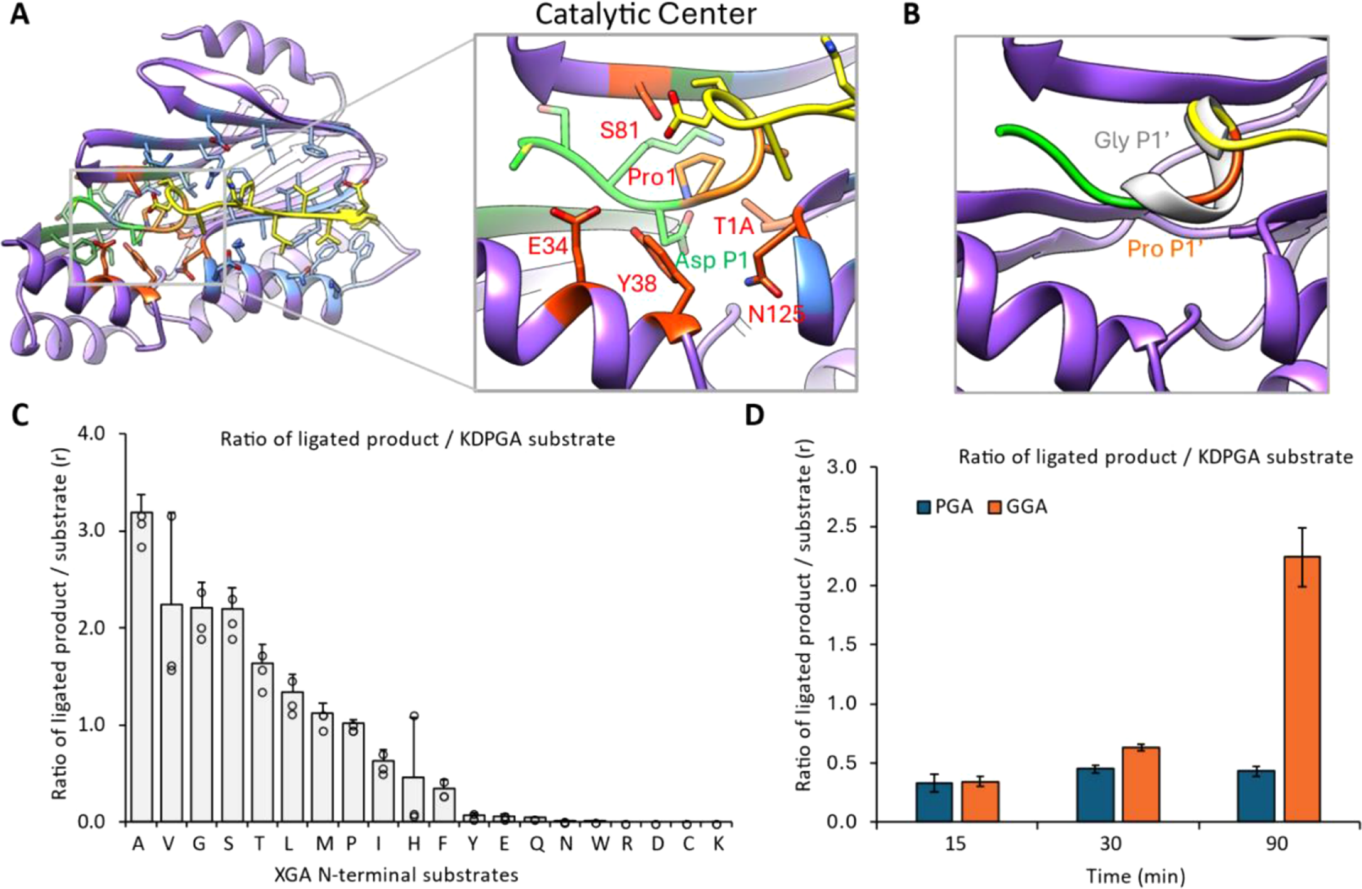
Structure-based modifications of the N-terminal recognition motif of mmConnectase rendering an irreversible protein ligation activity. (A) The side view of the mmConnectase with a substrate peptide atomic model, together with a zoomed-in illustration of the c-terminal (green), n-terminal (yellow), and Pro–Gly–Ala catalytic pocket (orange) of the substrate within the mmConnectase recognition groove. (B) Atomic model illustrating the Pro–Gly–Ala substrate motif is under high constraint within the mmConnectase recognition groove; by switching this residue to smaller amino acids (Gly–Gly–Ala in dark gray), a new stable α-helical secondary structure could be formed, which is less likely to be cleaved. (C) Screening of first amino acid residue at the N-terminal recognition motif where various XGA N-terminal substrates were used. (D) Time-based ligation assay comparing two prominent N-terminal substrates: PGA and GGA. The MS analysis of the ligations was determined, and measurement was calculated based on the ratio of the ligated product against C-terminal KDPGA substrates (Figure S3). The assays are performed as *n* = 3 independent assays, and the graph bars represent the mean with standard deviation (SD) and the circles represent individual data.

During either a protease or protein ligation reaction, the C-terminal substrate must sit in the catalytic pocket of MmCET. As shown in Figure 2A, the peptide bond after the aspartic acid residue at the P1 position could be attacked by threonine 1 from MmCET (note that the T1A in the actual crystal structure renders the enzyme inactive), forming a transition state complex in which the C-terminal substrate is directly linked to Thr1 of MmCET via a peptidyl bond, while proline at the P1’ position is cleaved. This reaction requires destabilization of the peptidyl bond between Asp at P1 and Pro at P1’ to be energetically favored. Indeed, by observing the conformation of a peptide substrate captured in our complex structure, we noticed that the Pro-Gly-Ala sequence at the P1′–P3′ positions is highly unstable (Figure 2B, orange color) based on its bond angle constraints. Thus, we would like to understand how a less stressed amino acid at P1’ would impact MmCET’s processivity. We then prepared 20 different peptide ligands with different amino acids at the P1’ position at its very N-terminal. So, when they were consumed during the first round of ligation, a more stabilized P1’ residue would prevent the ligation product from repeated cleavage. Interestingly, smaller amino acids, like Gly or Ala, etc., at the P1’ position significantly improve the eventual peptide ligation efficiency (Figure 2C, as measured by the product/substrate ratio using mass-spec, Figure S7). Indeed, if we attempt to dock a Gly residue at P1’ using Coot, the resulting structure showed a relatively stable secondary structure with minimal constraints at the P1–P1’ junction (shown in Figure 2B, gray color). Interestingly, validating this hypothesis helped us to obtain a much more processive protease as we describe in the next section.

### Structure-Based Modification of Substrate Recognition Motifs to Suppress Reversible Protein Ligation

To uncover the source of this enhancement, we conducted a series of time-course ligation experiments, again monitoring the output/input ratio (Figure 2D). Gly–Gly–Ala was significantly more processive than Pro–Gly–Ala. This is because, in the ligated products, internal peptide sequences can be recognized by MmCET. If Pro-Gly-Ala remains at the P1′–P3′ positions, the ligated product can be easily cleaved and reused as a substrate. In the case of the N-terminal GGA peptide, once ligated with the C-terminal substrate, Gly–Gly–Ala in the catalytic pocket is stable and the peptidyl bond between Asp (P1) and Gly (P1’) is not stressed, preventing further cleavage. After 30 min, the product and substrate for the PGA design reach equilibrium, whereas the GGA design allows the ligation reaction to continue, consuming the substrate and accumulating the products. Thus, using a GGA N-terminal substrate or similar residues that better fit the catalytic pocket conformation makes the MmCET-catalyzed ligation reaction irreversible and highly processive.

This finding overcomes a major disadvantage of MmCET and provides a potential strategy to enhance repurposed proteases as efficient protein ligases.

### Applications in Protein Ligation Experiments

**Figure 3:**
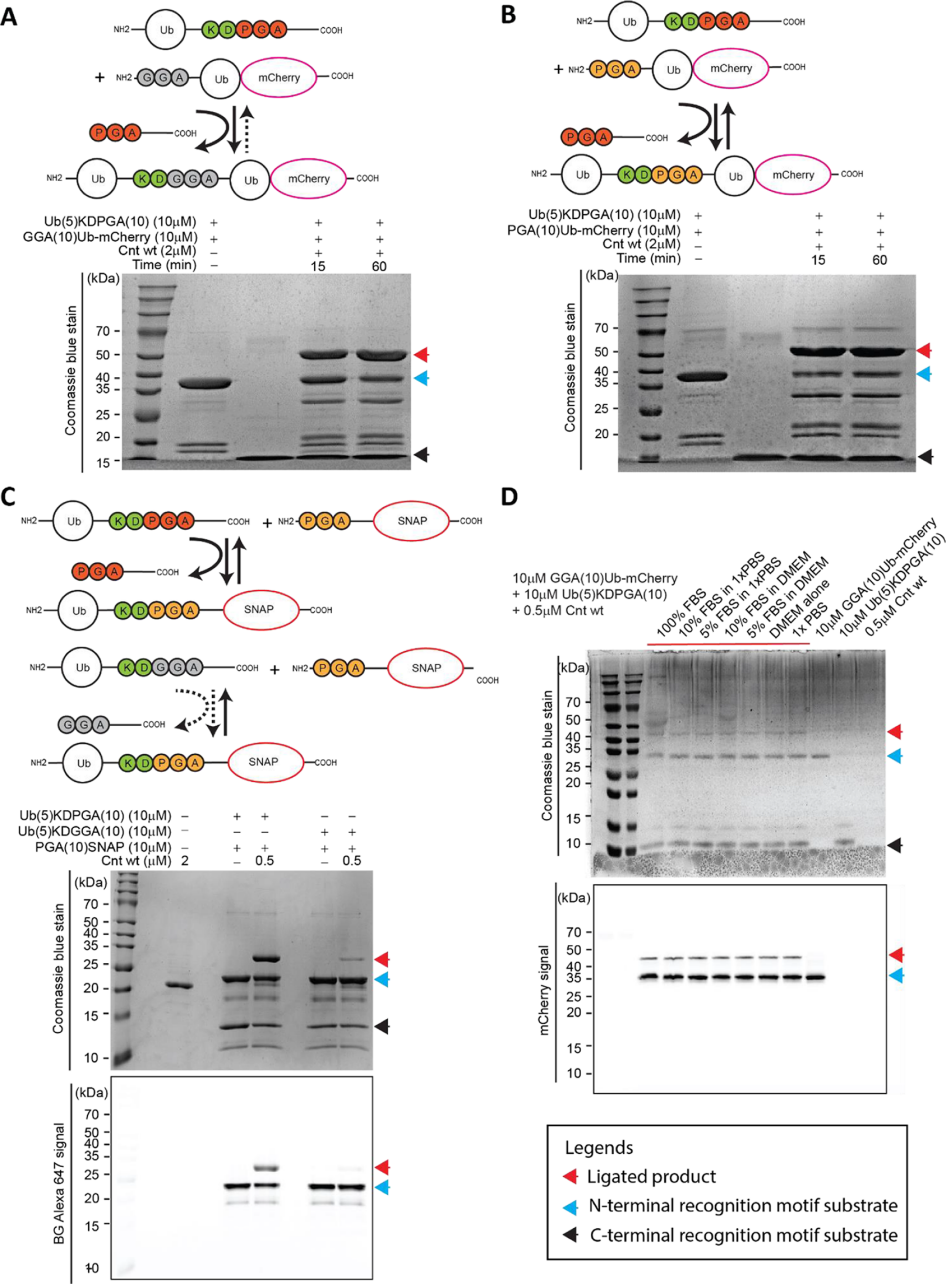
Ligase activity and buffer compatibility of mmConnectase using well-folded proteins as substrates. (A) Schematic representation of the irreversible ligation activity of GGA N-terminal substrates with the KDPGA C-terminal substrate. SDS-PAGE results demonstrating the good yield of the final product and ligation achieved by mmConnectase at 15 and 60 min reaction times. (B) Schematic representation of the reversible ligation activity of PGA N-terminal substrates with the KDPGA C-terminal substrate. SDS-PAGE results demonstrating the poor yield of the final product and ligation achieved by mmConnectase at 15 and 60 min reaction times. (C) Schematic representation of the weak cleavage of the KDGGA C-terminal substrate as compared with the KDPGA C-terminal substrate. SDS-PAGE results demonstrating the yield of the final product and ligation achieved by mmConnectase with different C-terminal substrates. (D) Determination of the compatibility of various cellular culturing medium conditions with the ligation efficiency of Cnt wt. SDS-PAGE results demonstrating the ligation of two substrates denoted with black arrows: Ub(5)KDPGA(10) and GGA(10)ub-mCherry in various conditions as indicated. Ligated product is denoted with red arrows.

Bearing in mind the GGA-enhanced processivity for the peptide substrates described above, we designed protein ligation experiments to test whether our findings hold true for large, well-folded protein substrates. First, we confirmed that substrates with a C-terminal motif containing Pro–Gly–Ala at the P1′–P3′ positions are readily recognized and compatible with MmCET-catalyzed ligation using a Gly–Gly–Ala N-terminal motif (Figure 3A). 10 μM of the protein substrate (Ub-KDPGA and GGA-Ub-mCherry, respectively) was incubated with 2 μM MmCET, resulting in more than 40% (estimated based on the intensity) of the substrate being ligated after 15 min, with the ligation yield increasing to over 60% after 60 min. This demonstrated that a combination of the C-terminal KDPGA sequence and an N-terminal GGA motif is highly effective in MmCET-catalyzed protein–protein ligation experiments.

To verify whether the processivity of MmCET is indeed improved by swapping to the GGA N-terminal motif, we performed the same ligation experiment using a combination of C-terminal KDPGA and N-terminal PGA motifs. The ligation yield stagnated at approximately 40%, perfectly depicting the equilibrium state of a reversible ligation reaction (Figure 3B). To further confirm that the enhanced processive protein ligation is due to the cleavage-resistant property of a C-terminal or internal GGA at the P1′–P3′ location, we engineered two ubiquitin constructs with C-terminal KDPGA and KDGGA, respectively. We attempted ligation reactions with these two different C-terminal substrates side-by-side and tracked the ligation product using a fluorescently labeled SNAP N-terminal substrate (Figure 3C). The results confirmed our hypothesis and demonstrated that C-terminal KDGGA is highly resistant to further cleavage and reversible protein ligations. Thus, our structural analysis of the MmCET-substrate complex identified a method to enhance the processivity of MmCET-catalyzed protein ligations.

With this knowledge, we tested whether these modified substrate recognition motifs would facilitate efficient target protein modifications in a highly heterogeneous background, including under serum conditions. Typically, OaAEP1(C247A) ligation reactions were to be conducted in relatively clean environments, as the background peptide and protein environment, including the presence of occasional GL residues or similar combinations of N-terminal amino acids in the serum or at the surface of the cells, might participate in the labeling reaction (Figure S9), while serum-compatible sortase A requires a higher working concentration, usually around 20 μM.^25−29^ We conducted a series of protein–protein ligations in the presence of FBS of up to 100% (Figure 3D). MmCET demonstrated little reduction in efficiency, precisely recognizing Ub-KDPGA and GGA-Ub-mCherry, and ligating them smoothly. Furthermore, cellular surface labeling in the FBS-containing cell culture media using various concentrations of MmCET (0.05 to 2 μM) and various concentrations of GGA-SNAP (0.5 to 20 μM) demonstrated efficient and precise labeling of the cellular surface (Figures S10–S13). These data demonstrated the promising *in cellulo* compatibility of the structure-inspired enhanced MmCET ligation protocol.

### High-Precision Single Molecule Experiments Enabled by Enhanced MmCET Catalyzed Protein Ligation

In AFM-SMFS studies of protein, the target protein is immobilized in the system and polymerized proteins (polyprotein) are often constructed for precise single-molecule measurement, whose unfolding leads to characteristic sawtooth-like force–extension curves as reliable signals.^30−35^ To demonstrate the potential of connectase for robust protein immobilization and polymerization, we combined it with OaAEP1(C247A) for stepwise protein ligation using ubiquitin (Ub) and the 27th Ig domain (I27) of titin.^36^ The two protein monomers with specific enzymatic recognition peptide sequences on both termini, GL-Ub-(5)KDPGA(10) and GGA(10)-I27-NGL, were constructed for enzymatic ligation.

**Figure 4:**
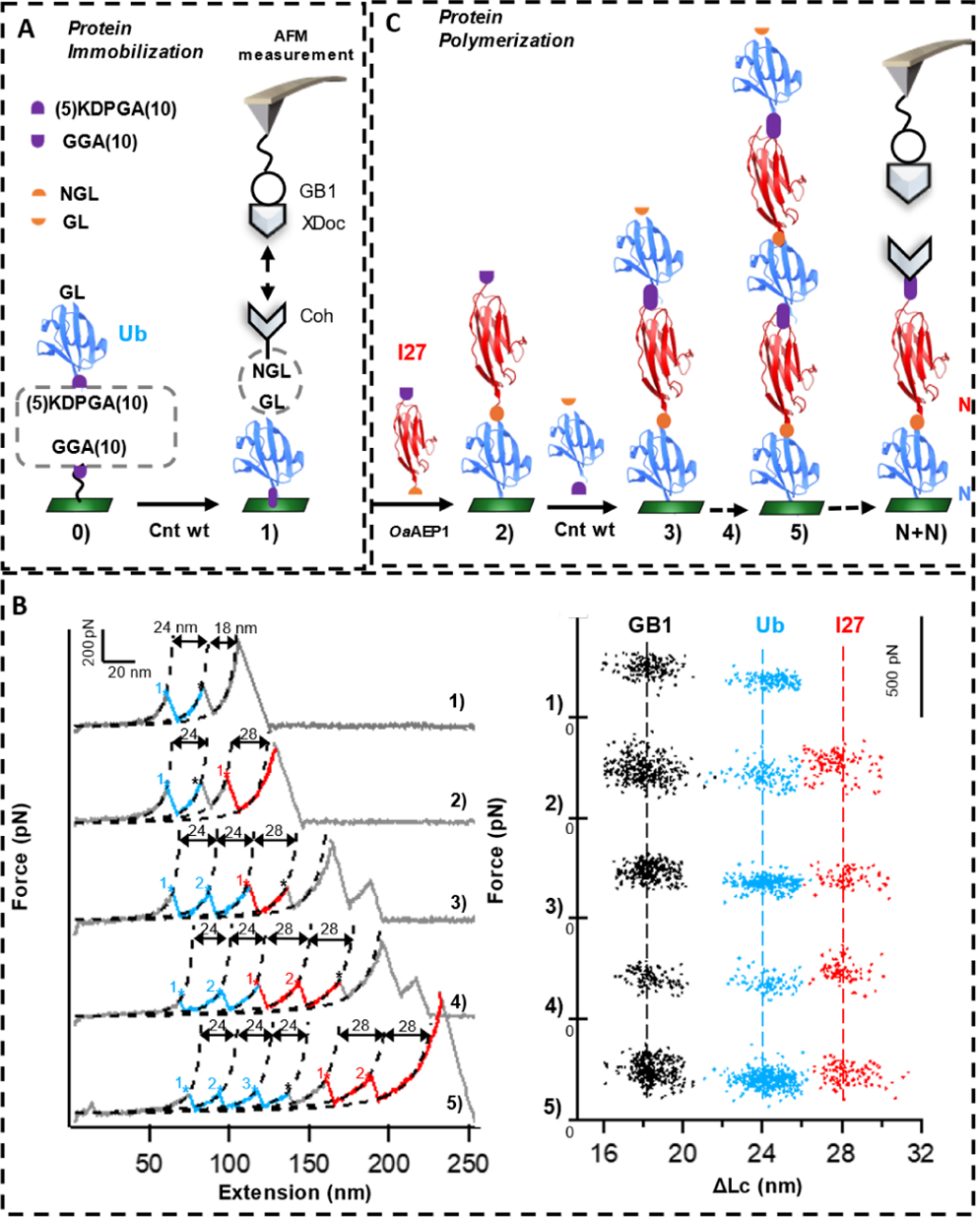
Protein immobilization and polymerization with controlled sequence using connectase and OaAEP1 verified by AFM-SMFS. (A) Ubiquitin (Ub) is immobilized on a glass surface by connectase, as verified by AFM-SMFS protein unfolding experiments. The GL-Ub-(5)KDPGA(10) protein is first ligated onto a GGA(10)-functionalized surface using connectase first (step 0) and then capped with Coh-NGL using OaAEP1 (step 1) between GL and NGL for precise AFM measurement with a GB1-XDoc-modified AFM tip. (B) Left panel: representative force–extension curves showing the expected number and type of protein unfolding peaks from the immobilized (poly)protein, including Ub (contour length increment ΔLc of 24 nm, blue), I27 (28 nm, red), GB1 (18 nm, black), and a final peak from the dissociation of the Coh–XDoc complex. Curves 1–5 correspond to (poly)proteins (Ub)1, (Ub)1-(I27)1, (Ub)2-(I27)1, (Ub)2-(I27)2, and (Ub)3-(I27)2, respectively. Right panel: the corresponding scatter plot showing the relationship between the unfolding force and ΔLc for GB1, Ub, and I27. For clarity, the force value is shifted by 500 pN for each sample. (C) Stepwise protein ligation using Connectase and OaAEP1 to build polyproteins with controlled sequence.

First, we immobilized Ub on a glass surface using connectase (Figure 4A). Peptide GGA(10)-N3 was coated on a DBCO-functionalized glass coverslip via click chemistry as previously reported (Supporting Information).^37^ Then, the ubiquitin monomer GL-Ub-(5)KDPGA(10) was immobilized on the coverslip catalyzed by connectase (step 0). For precise AFM-SMFS measurement, Coh-NGL was reacted with Ub via OaAEP1 between GL and NGL peptide sequences as a pulling handle (step 1), and an AFM tip functionalized with GB1–XDoc was used to pick up the Ub via reversible Coh–XDoc interaction.^38,39^ Upon stretching, the representative force–extension curve showed two unfolding peaks (Figure 4B, curve 1 and plot 1), corresponding to Ub (contour length increment from protein unfolding ΔLc, ∼24 nm) and unfolding fingerprint GB1 (∼18 nm) with normal unfolding force,^40^ respectively, confirming successful protein immobilization.

Next, we demonstrated the stepwise ligation of the protein on the surface. After Ub immobilization, we skipped ligation (step 1) of Coh-NGL for direct AFM measurement. Instead, the GGA (10)-I27-NGL monomer was ligated first using OaAEP1, leading to the construction of (Ub)1-(I27)1 (Figure 4C, step 2). Then, Coh-NGL was capped for AFM measurement as before, which showed an additional unfolding peak (28 nm) from I27 as expected.

Following this strategy, we repeated this stepwise enzymatic ligation procedure (Figure 4C). As a result, polyproteins with controlled sequences, (Ub)2-(I27)1, (Ub)2-(I27)2, and (Ub)3-(I27)2, were all obtained through this method using the OaAEP1 and MmCET (steps 3–5). Their AFM-SMFS measurements all showed expected unfolding events from the Ub and I27. Thus, the result demonstrated that our connectase is an excellent enzyme for protein immobilization and polymerization, which is bio-orthogonal with other enzymes.

## Conclusion

The identification of Connectase has significantly expanded the toolbox of protein ligases with a novel scaffold, opening new frontiers in biochemical research and applications. Like plant AEPs, this enzyme family was initially recognized for its protease activity.^41^ Our research reveals that the key to transforming proteases into highly efficient ligases lies in the precise modification of critical structural features. By determining the crystal structure of MmCET, both with and without a preferred peptide substrate at its catalytic transition state, we uncovered the essential elements responsible for the high-precision substrate recognition of N-terminal motifs.

Capitalizing on these newly characterized properties of MmCET, we have successfully addressed a major limitation of this enzyme, enhancing its processivity and demonstrating its superior performance in tandem protein ligation through single-molecule experiments. This breakthrough not only showcases the potential of Connectase but also paves the way for applying similar structure-based engineering strategies to other protease scaffolds.^42^

The insights gained from our study bring us closer to designing tailor-made, highly specific protein ligase tools for a wide range of biological applications.^43,44^ The versatile structural features of MmCET, particularly its highly adaptable N-terminal recognition groove, promise unprecedented opportunities for specificity engineering, opening ways to bioorthogonal ligations. Mastering the specificity of substrate recognition in MmCET and related protein ligases will enable the development of next-generation protein ligases with unparalleled precision and efficiency.

## Supporting Information Available

Figures S1–S13; experimental methods and materials; crystallographic data and statistics (Table S1) (PDF)

## Abbreviations

AEPs: asparaginyl endopeptidases
CETs: connectases
AFM-SMFS: atomic force microscopy-based single-molecule force spectroscopy
Ub: ubiquitin
FBS: fetal bovine serum
