# Supplementary material for: Structural Basis of High-Precision Protein Ligation and Its Application
Source: J Am Chem Soc. 2025 Jan 2;147(2):1604–11. doi: 10.1021/jacs.4c10689 (PMC11745163; doi:10.1021/jacs.4c10689)
Supplement: Supplementary file 1 — ja4c10689_si_001.pdf [file ja4c10689_si_001.pdf]

Supplementary information

Structural Basis of High Precision Protein Ligation And Its Application

Kelvin Han Chung Chong[1, 2#], Lichao Liu[3#], Rae Chua[2, 4], Yoke Tin Chai[1], Zhuojian Lu[3], Renming Liu[3], Eddie Yong Jun Tan[1], Jinxi Dong[1], Yek How Khoh[1], Jianqing Lin[1, 2], Franklin L. Zhong[4,5], Julien Lescar[1, 2], Peng Zheng[3*], Bin Wu[1,2*]

[1]School of Biological Sciences, Nanyang Technological University, 60 Nanyang Drive, Singapore 636921.

[2]NTU Institute of Structural Biology, Nanyang Technological University, EMB 06-01, 59 Nanyang Drive, Singapore 636921.

[3]State Key Laboratory of Coordination Chemistry, Chemistry and Biomedicine Innovation Center (ChemBIC), School of Chemistry and Chemical Engineering, Nanjing University, Nanjing, 210023, P. R. China

[4]Lee Kong Chian School of Medicine, Nanyang Technological University, 308232 Singapore.

[5]Skin Research Institute of Singapore (SRIS), 308232 Singapore.

Table of Contents

Figure S1 multiple sequence alignment and taxonomy of connectase from various archaeal species

Figure S2. Manual fitting of the peptide substrate residues.

Figure S3. mmConnectase mutant panel – well folded protein ligation

Figure S4. mass-spec spectrums of substrate and product peptides

Figure S5. mass-spec spectrums of alanine substitution at relevant position of substrate and product peptides

Figure S6. graph plot of ligated product peptides against alanine scanning substrate at relevant positions

Figure S7. mass-spec spectrum of XGA N-terminal substrate and product peptides

Figure S8. mass-spec spectrums of time-based PGA/GGA substrate and product peptides

Figure S9. OaAEP1 (C247A) non-specifically cross react with intrinsic surface receptors on cell lines.

Figure S10. Specific cellular surface labelling on HEK293T cells by Connectase (mmCET).

Figure S11. Cellular surface labelling on HEK293T cells by Connectase (mmCET) with various concentration of substrate and ligase.

Figure S12. Flow cytometry analysis to validate cellular surface ligation of Connectase (mmCET) in HEK293T cells.

Figure S13. Precise protein ligation on cellular surface conducted by mmCET.

Table S1. X-ray Crystallography data collection and refinement statistics

Experimental Methods

Table S2. Nucleotides and Proteins sequence list

Table S3. Plasmids sequence

Table S4. Peptides list

A

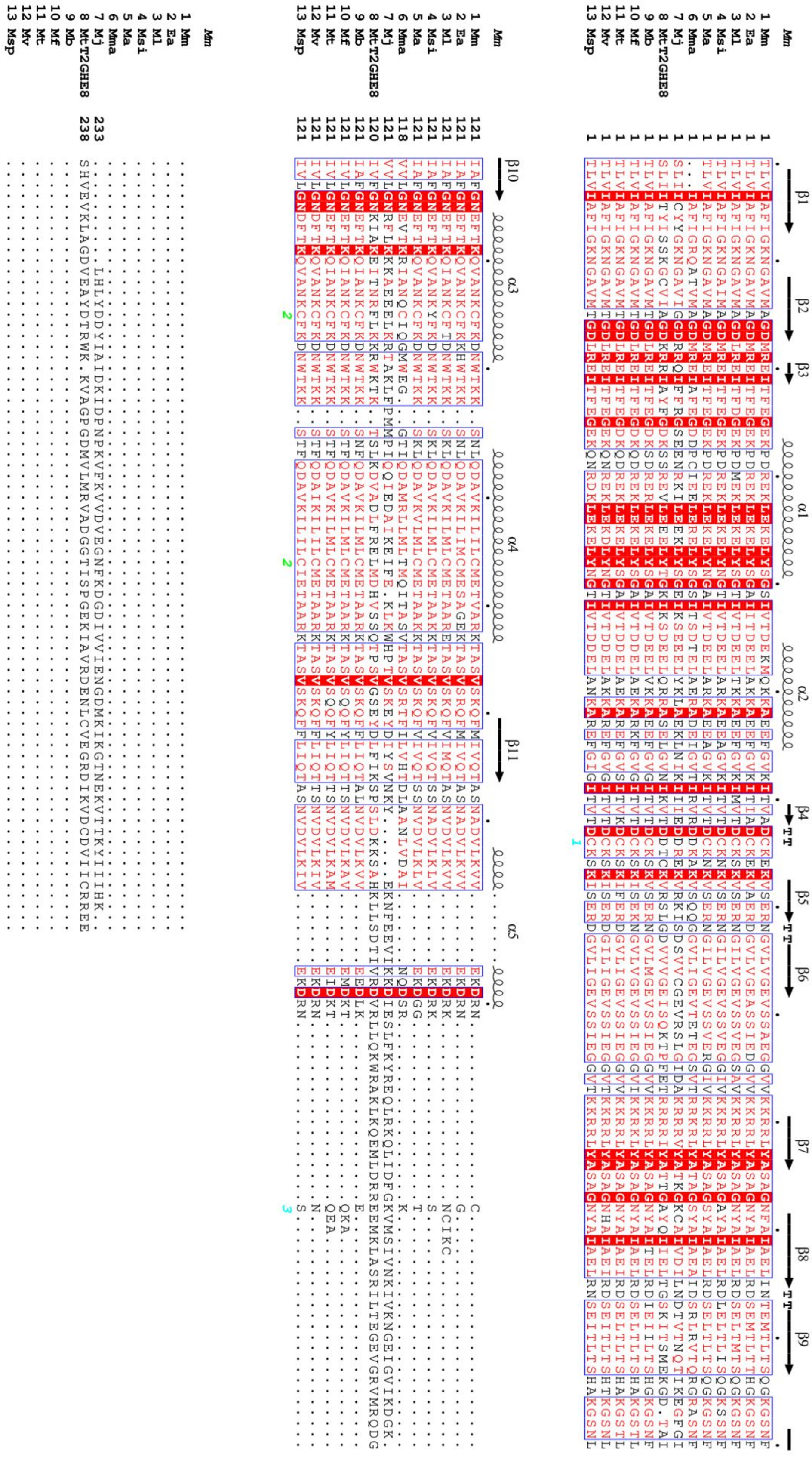

B

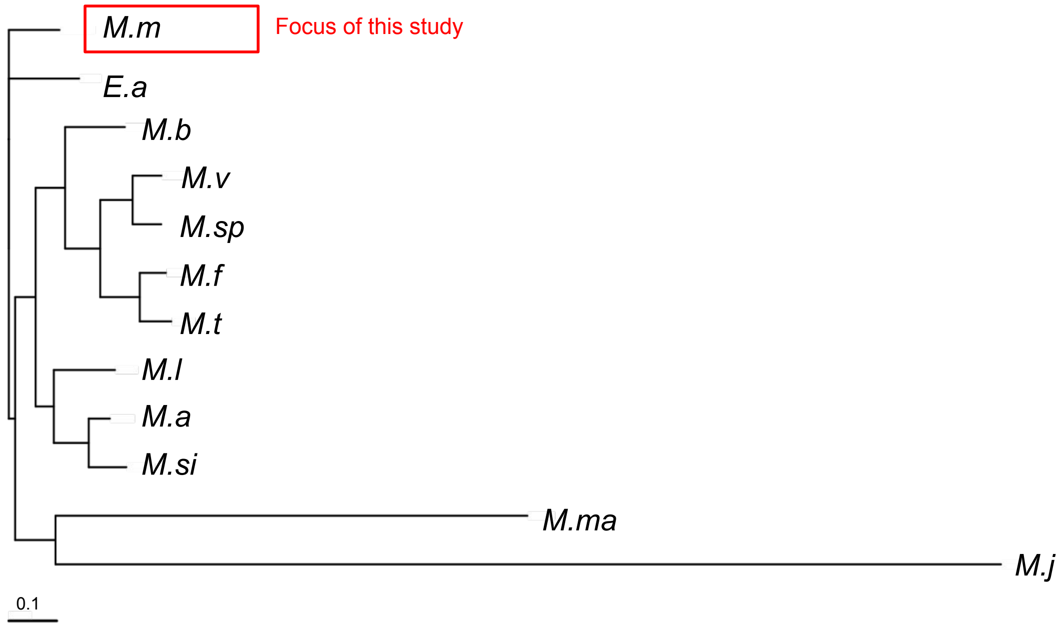

C

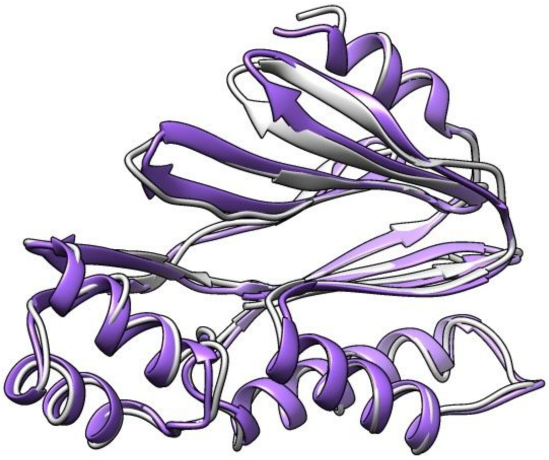

Apo form in light grey color, PDB 8JTU; peptide bound form in light purple, PDB 8WKD

D

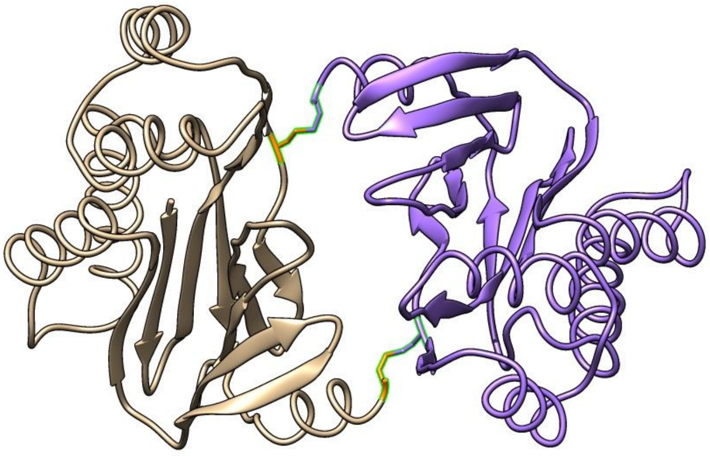

Cysteines in MmCET form stable disulfide bonds (C65-C193), locking the enzyme in a dimer conformation

E

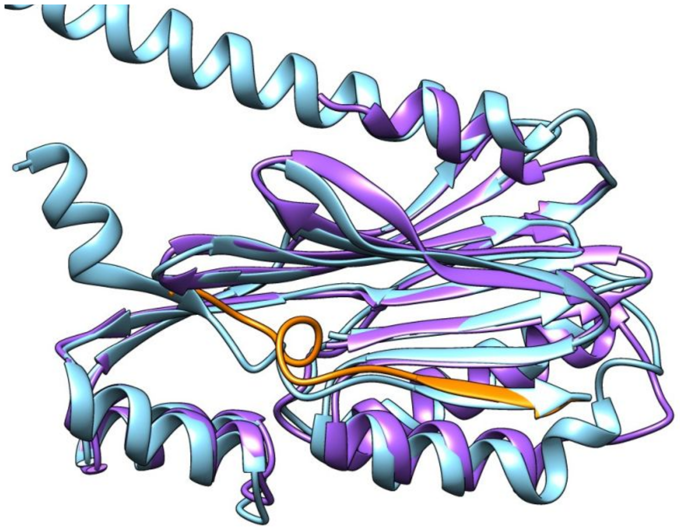

MmCET with peptide bound form in dark purple, PDB 8WKD; MjCET with substrate derived peptide bound in blue, adopted from PDB 6ZW0

Supplementary figure 1. Multiple sequence alignment and taxonomy of connectase from various archaeal species. A) multiple sequence alignment of various connectase from various archaeal species. B) Taxonomy classification of connectase from various archaeal species. C) Model illustrating both Apo and bound form of mmCET. Apo form in light grey color, PDB 8JTU; peptide bound form in light purple, PDB 8WKD. D) Model showing cysteines in MmCET form stable disulfide bonds locking the enzyme in a dimer conformation. E) Models showing mmCET with peptide bound form in dark purple, PDB 8WKD; mjCET with substrate derived peptide bound in blue, adopted from PDB 6ZW0

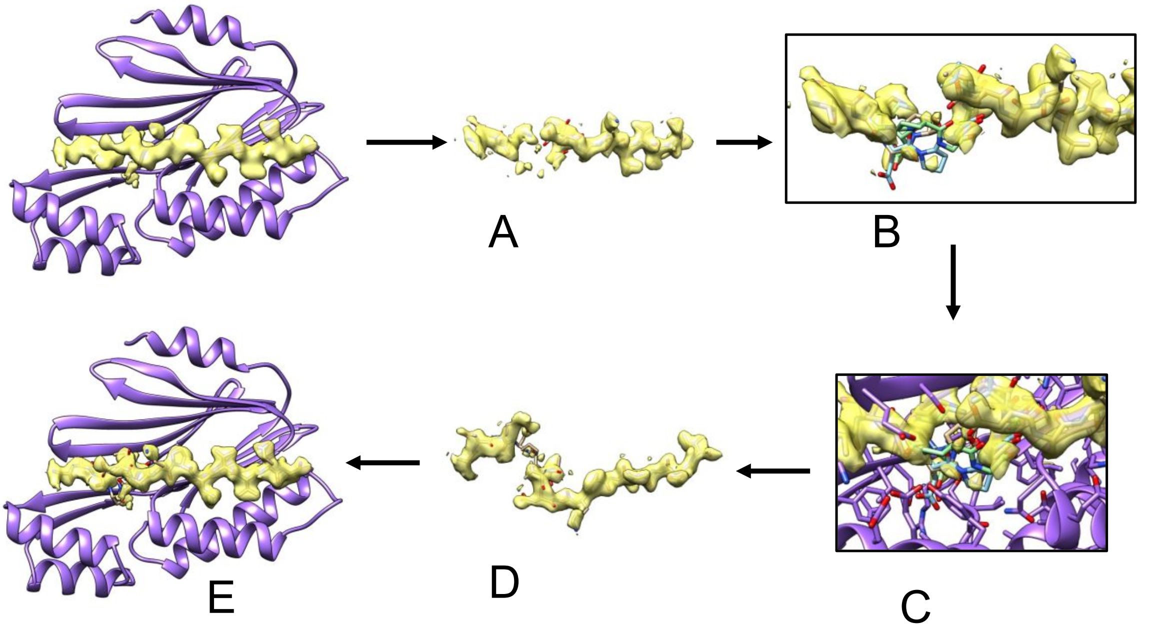

Supplementary figure 2. Manual fitting of the peptide substrate residues. The weak density observed at the P1′ and P2′ positions likely results from the averaging effect of highly flexible local conformations. Therefore, we subtracted the enzyme density to focus specifically on constructing the peptide model.

(A) When only the peptide substrate density was present, we began manually building the peptide, adding amino acids sequentially in Coot for residues with clearly defined side chains, leaving P1′ and P2′ positions unoccupied.

(B) We next constructed several plausible conformations for the P1′P2′ region and assessed them through molecular dynamics simulations in Amber, converging on three likely conformations.

(C) These peptide models were then placed back into the enzyme's substrate-binding pocket to evaluate their compatibility with the binding environment.

(D) The reported conformation for the P1′P2′ residues are the most probable arrangement, compatible with the proposed catalytic hydrolysis mechanism.

(E) Finally, the entire enzyme-peptide complex was refined against the density in Phenix to ensure structural accuracy.

A

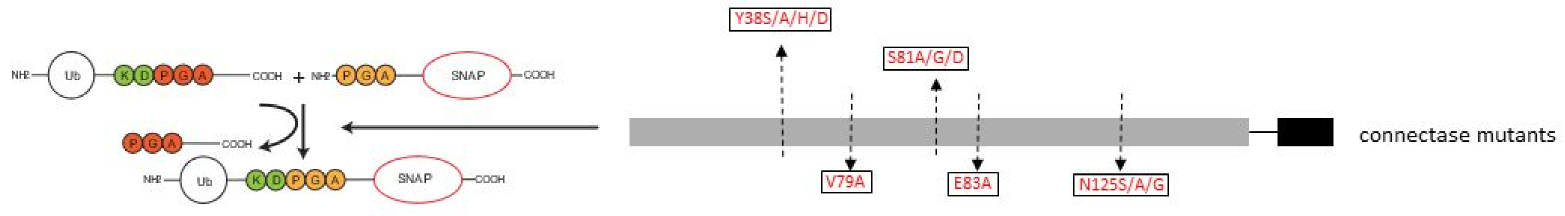

Connectase Amino acid sequence

TLVIAFIGKNGAVMAGDMREITFEGEKPDREKLEKELY^{38}SGSIVTDEEMQKKAEEFGVKITVADCKEKVSERNGVLVGEV^{79}SS^{81}AE^{83}GGVVKKRRLYASAGNFAIAELINTEMTLTSQGKGSNFIAFGN^{125}EFTKQVA NKCFKDNWTKKSNLQDAVKILILCMETVARKTASVSKQFMIVQTASNADVLKVVEKDRNC

B

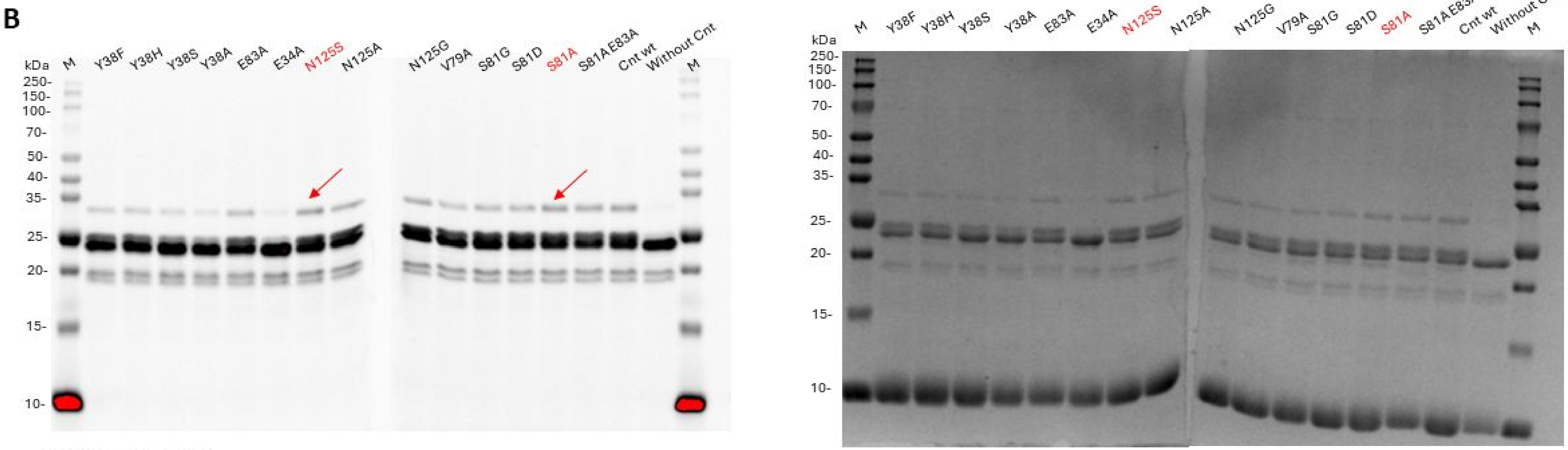

SNAP-BG647

Coomassie blue

Red Arrow denotes the improved efficacy for ligated product band

Supplementary figure 3. MmConnectase mutant panel – well folded protein ligation. Residues surrounding the catalytic pockets have impact on its catalytic activity, demonstrated by protein-protein ligation experiments.

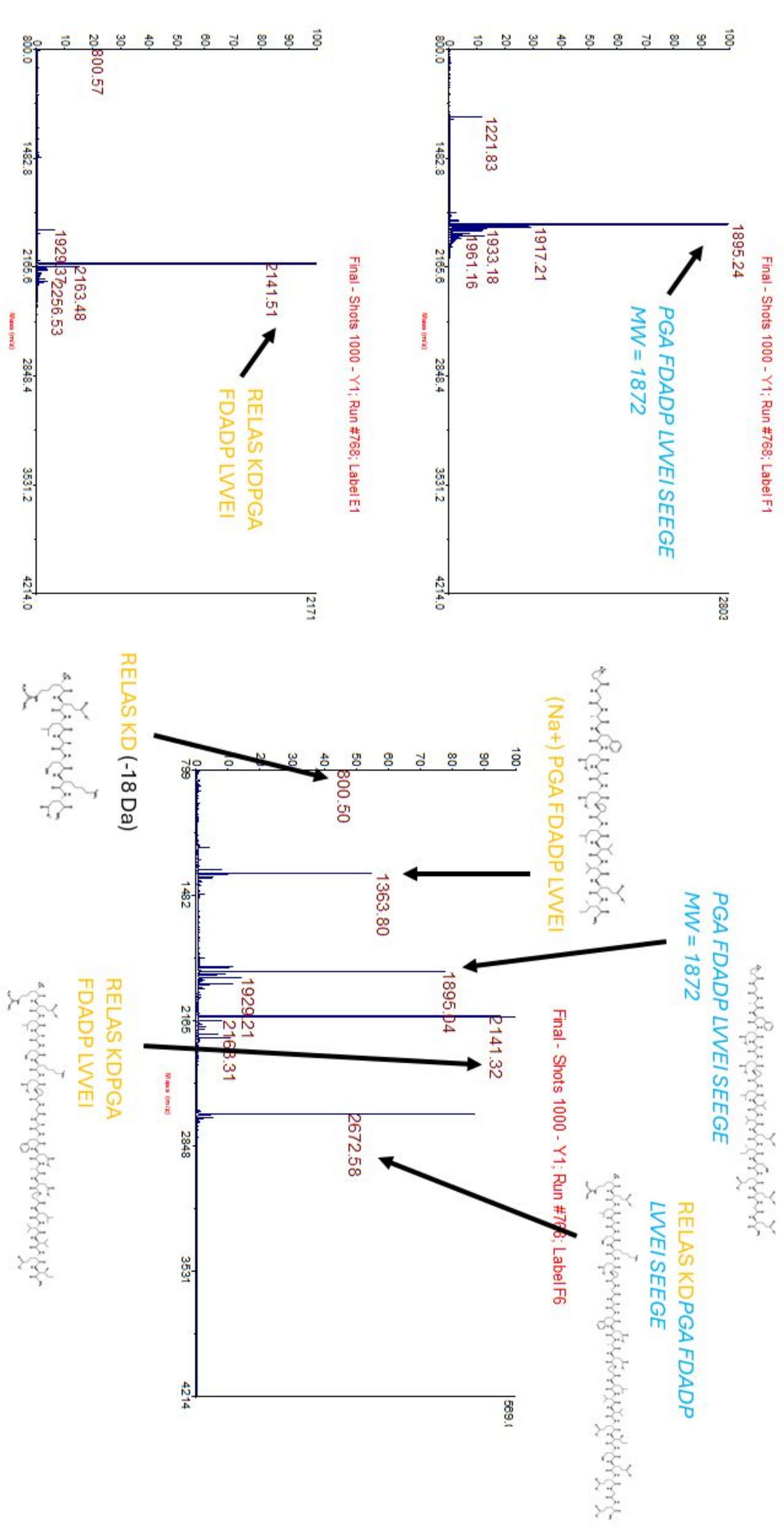

Supplementary figure 4. Mass-spec spectrums of substrate and product peptides. Illustration of the mass-spec results, explaining how we interpret the results and calculate the ‘apparent’ catalytic efficiency. The following charts are the raw data of the efficiency plots used in this manuscript.

Ctrl

PGA FDADP LVVEI SEEGE + RELAS KDPGA FDADP LVVEI

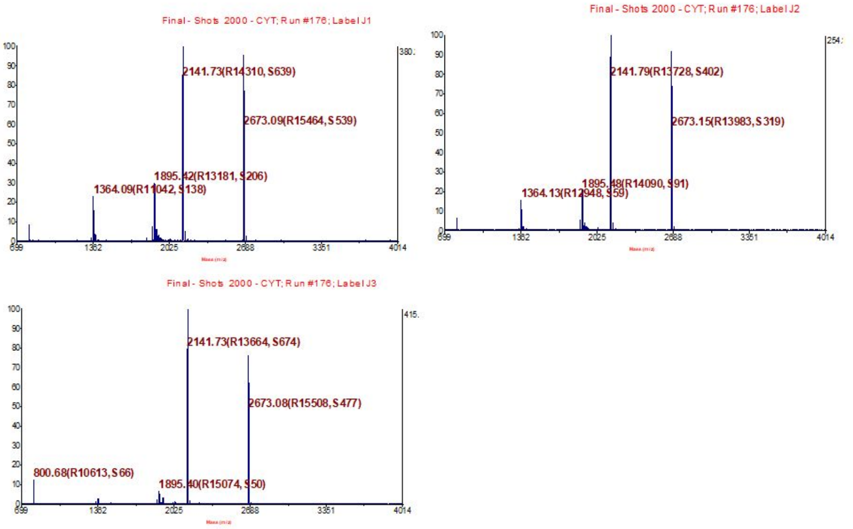

P7

PGA FDADP LVVEI SEEGE + AELAS KDPGA FDADP LVVEI

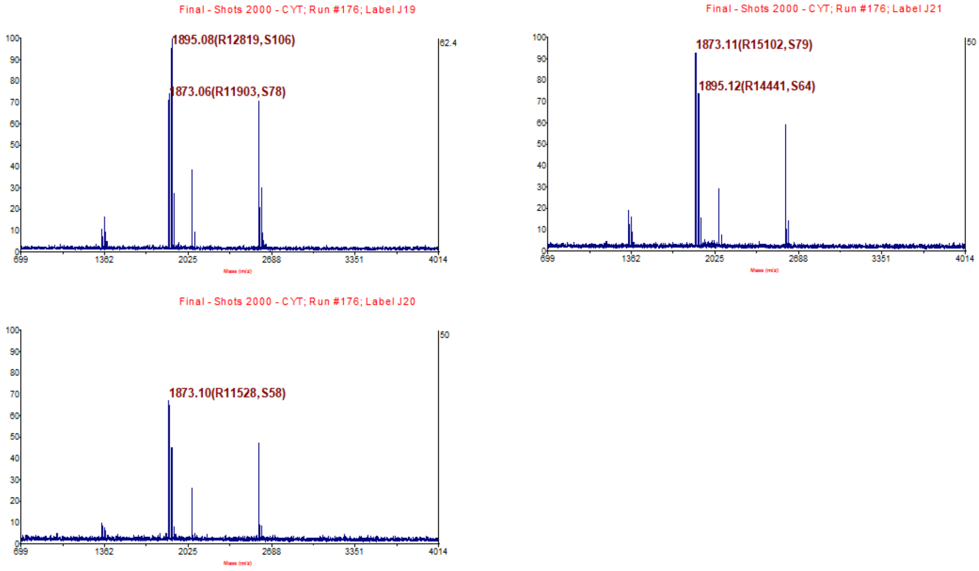

P6 PGA FDADP LVVEI SEEGE + RALAS KDPGA FDADP LVVEI

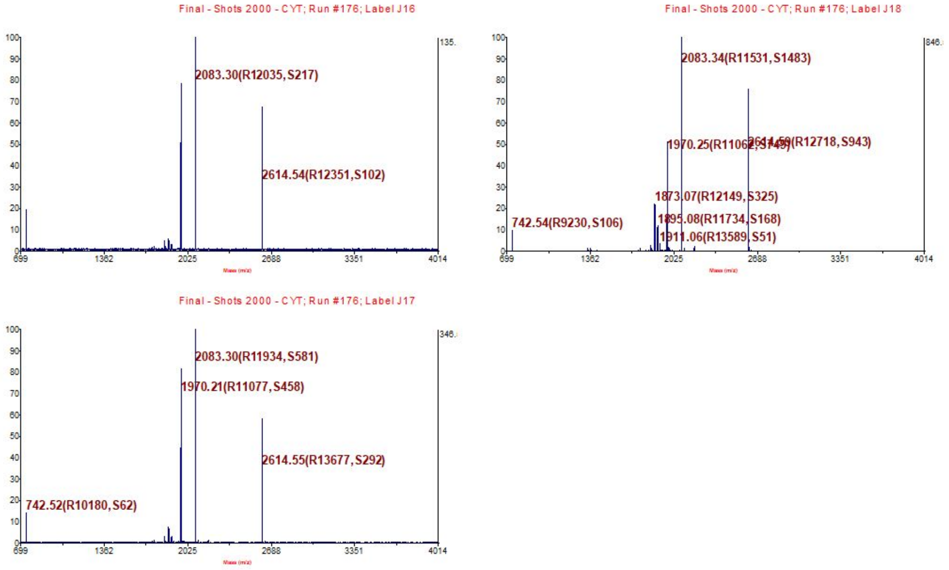

P5 PGA FDADP LVVEI SEEGE + REAAS KDPGA FDADP LVVEI

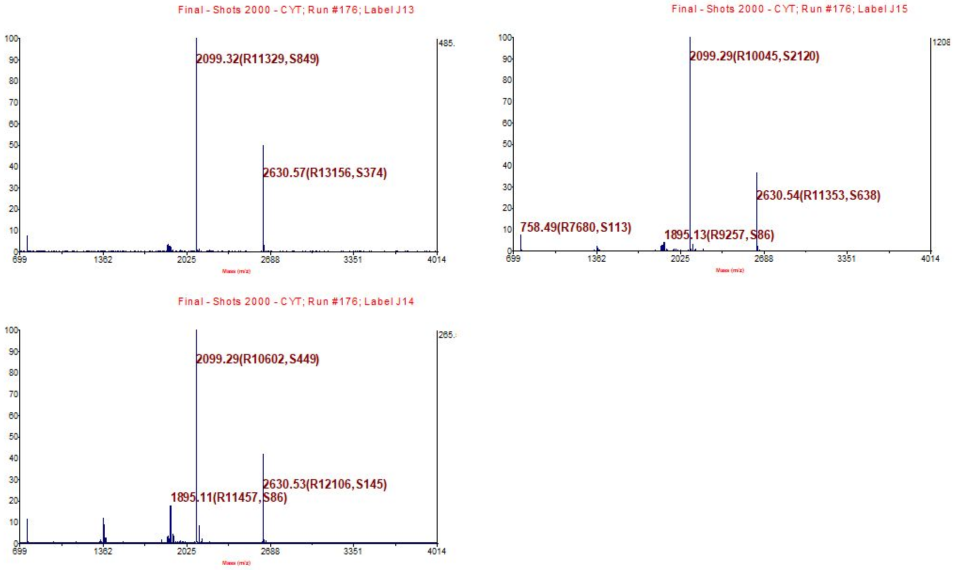

P3 PGA FDADP LVVEI SEEGE + RELAA KDPGA FDADP LVVEI

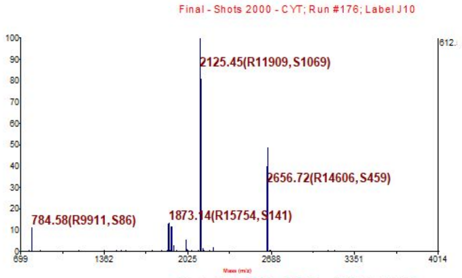

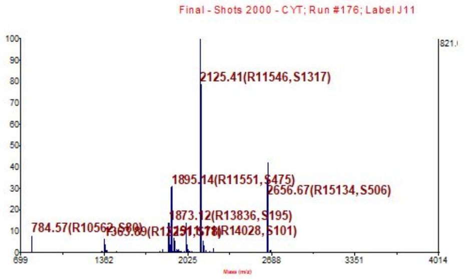

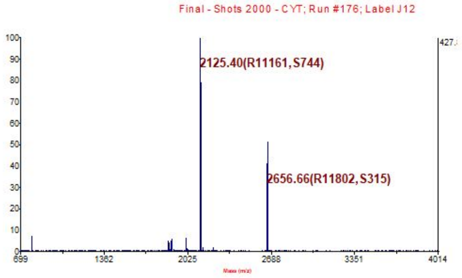

P2 PGA FDADP LVVEI SEEGE + RELAS ADPGA FDADP LVVEI

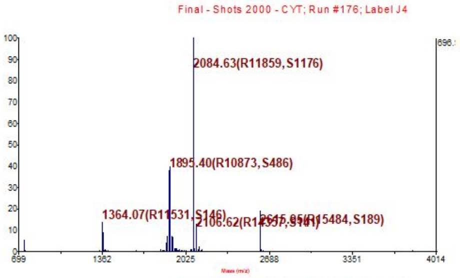

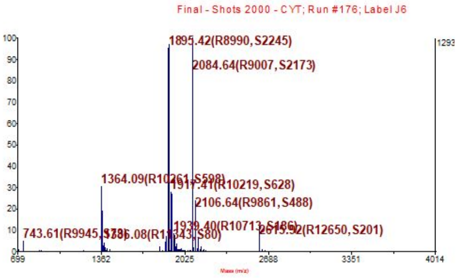

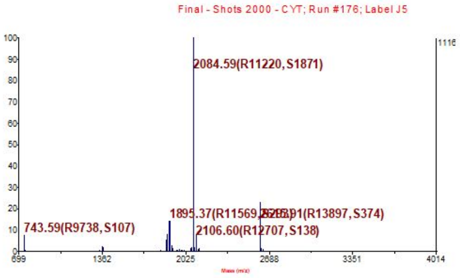

P4’ PGA ADADP LVVEI SEEGE + RELAS KDPGA FDADP LVVEI

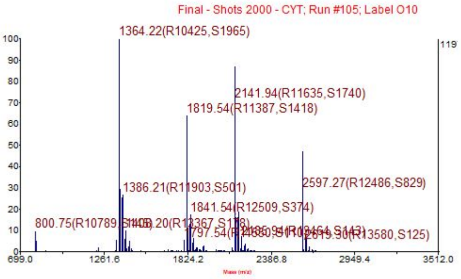

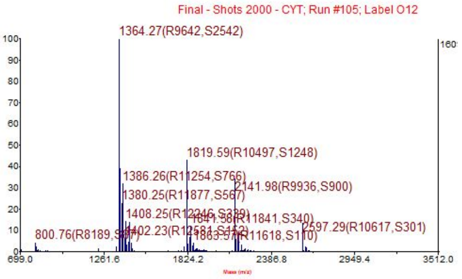

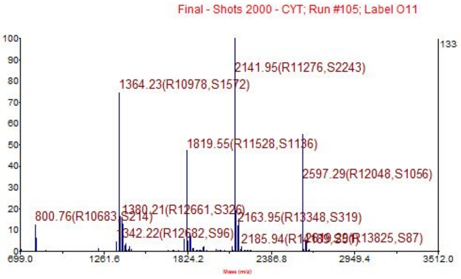

P5’ PGA FAADP LVVEI SEEGE + RELAS KDPGA FDADP LVVEI

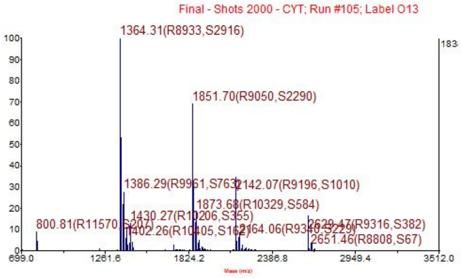

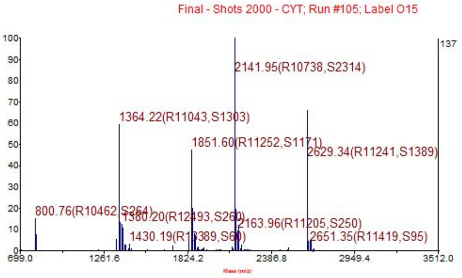

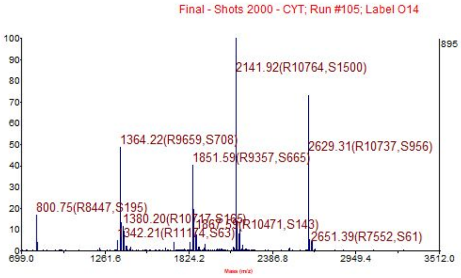

P7’ PGA FDAAP LVVEI SEEGE + RELAS KDPGA FDADP LVVEI

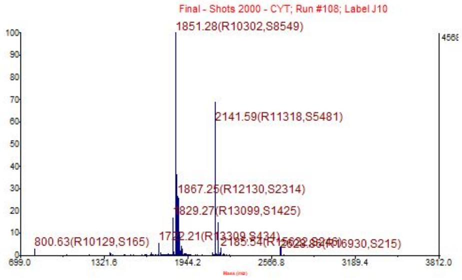

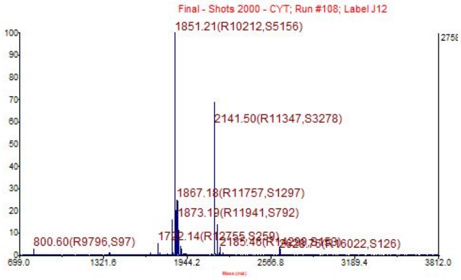

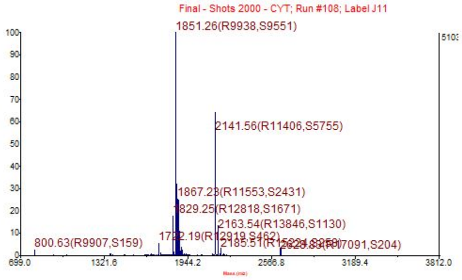

P8’ PGA FDADP LVVEI SEEGE + RELAS KDPGA FDADA LVVEI

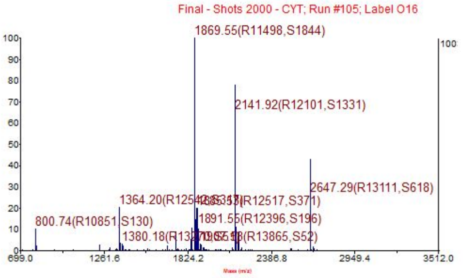

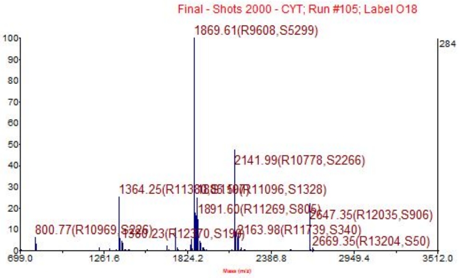

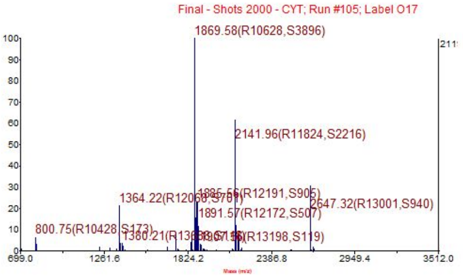

P9’

PGA FDADP AVVEI SEEGE + RELAS KDPGA FDADP LVVEI

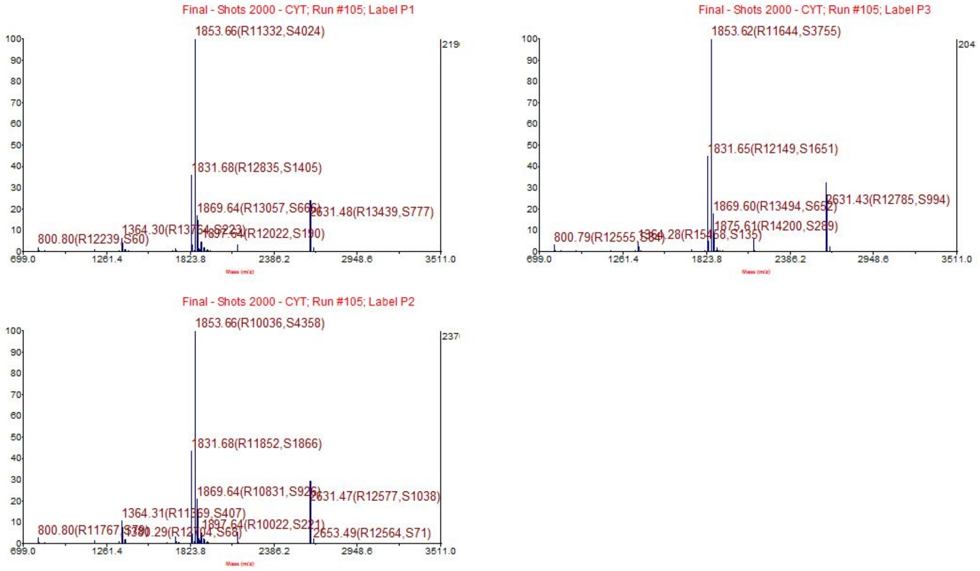

P10’

PGA FDADP LAVEI SEEGE + RELAS KDPGA FDADP LVVEI

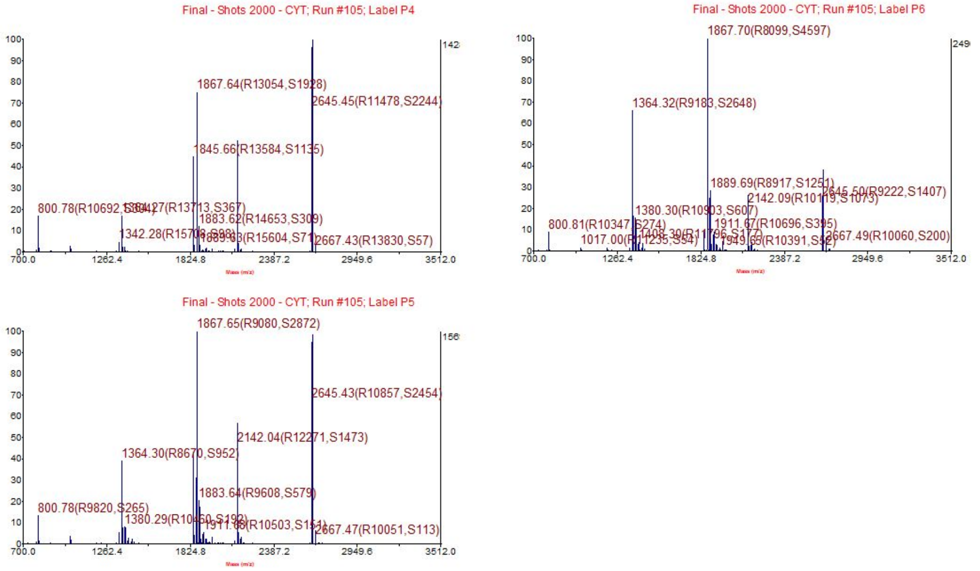

P11’ PGA FDADP LVAEI SEEGE + RELAS KDPGA FDADP LVVEI

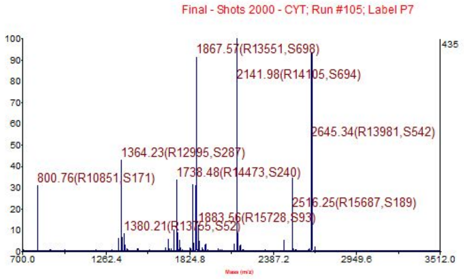

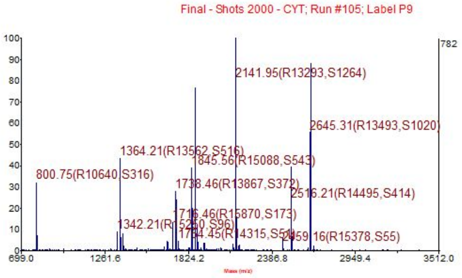

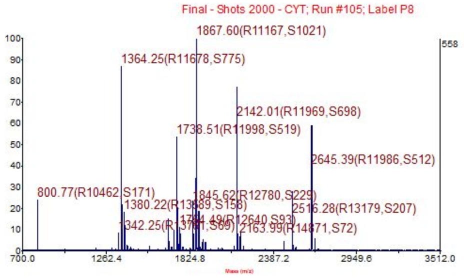

P12’ PGA FDADP LVVAI SEEGE + RELAS KDPGA FDADP LVVEI

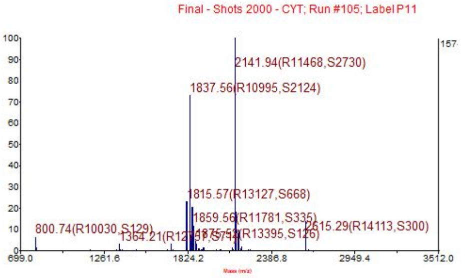

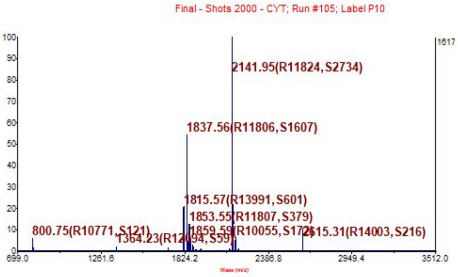

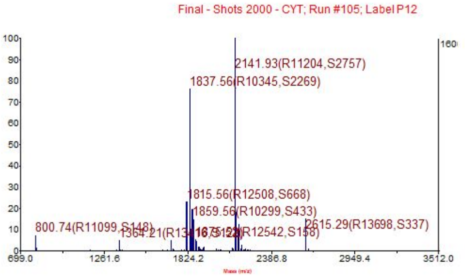

P13’ PGA FDADP LVVEA + RELAS KDPGA FDADP LVVEI

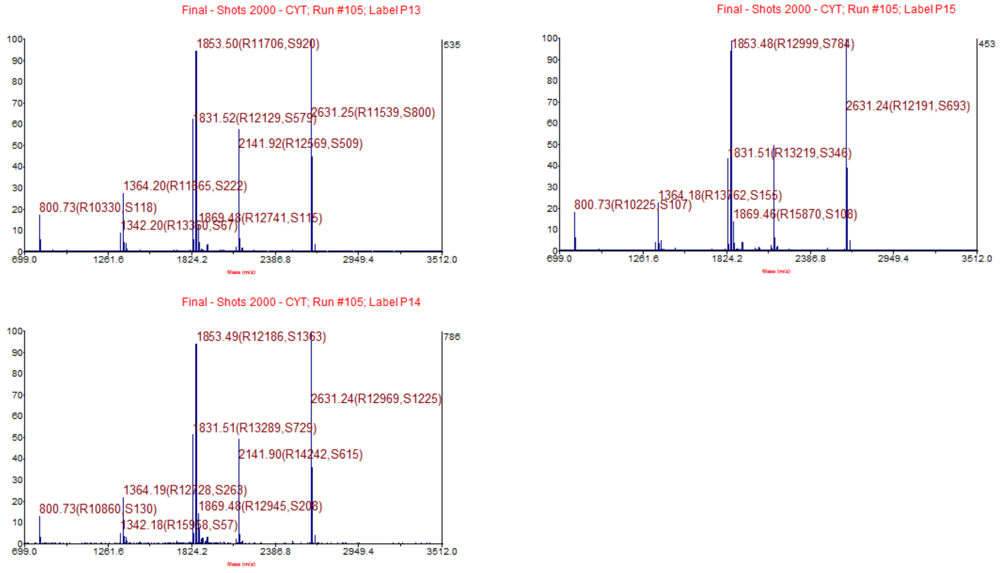

Supplementary figure 5. Mass-spec spectrums of alanine substitution at relevant position of substrate and product peptides

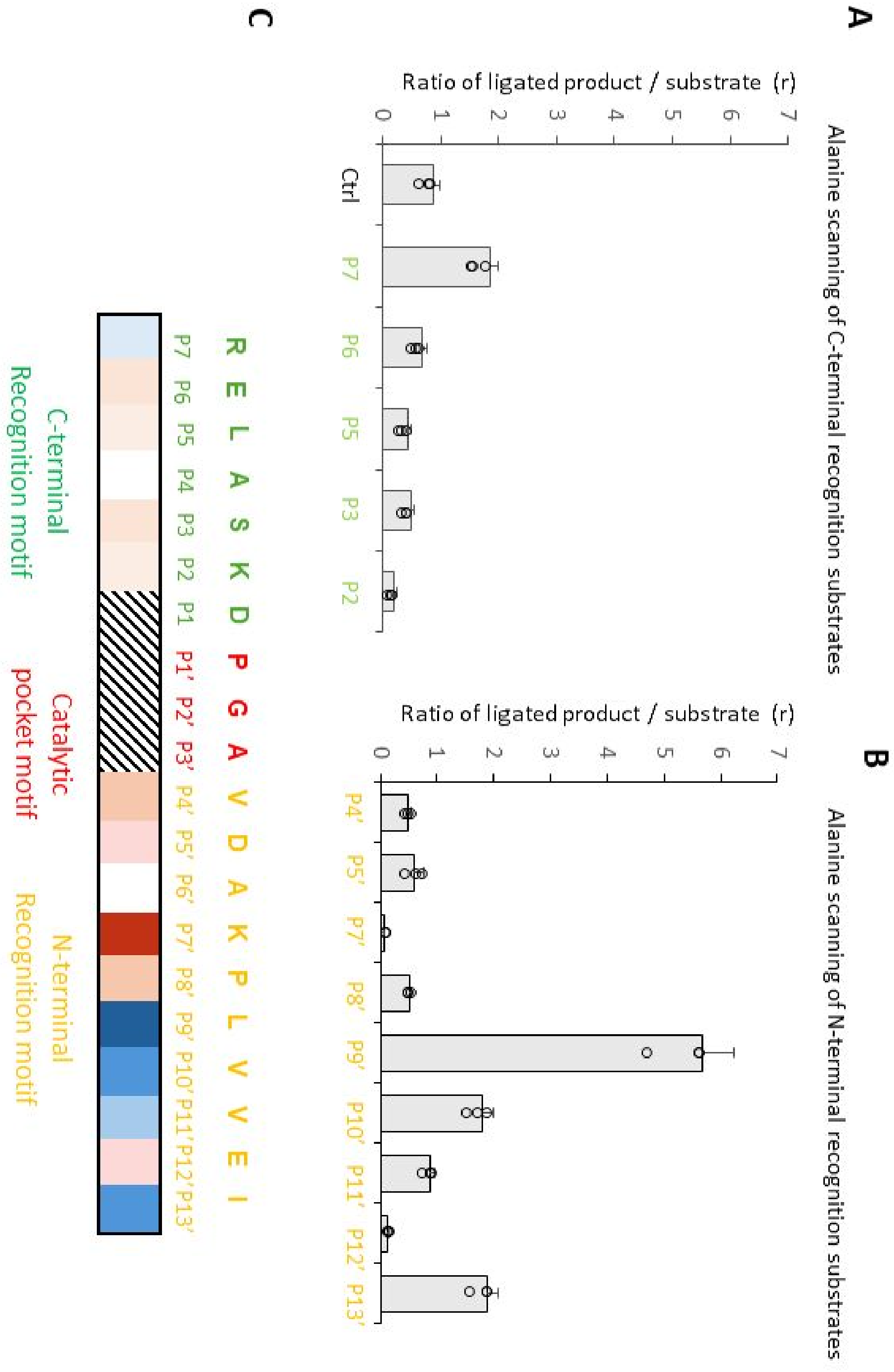

Supplementary figure 6. Graph plot of ligated product peptides against alanine scanning substrate at relevant positions

PGA15 5KDPGA10

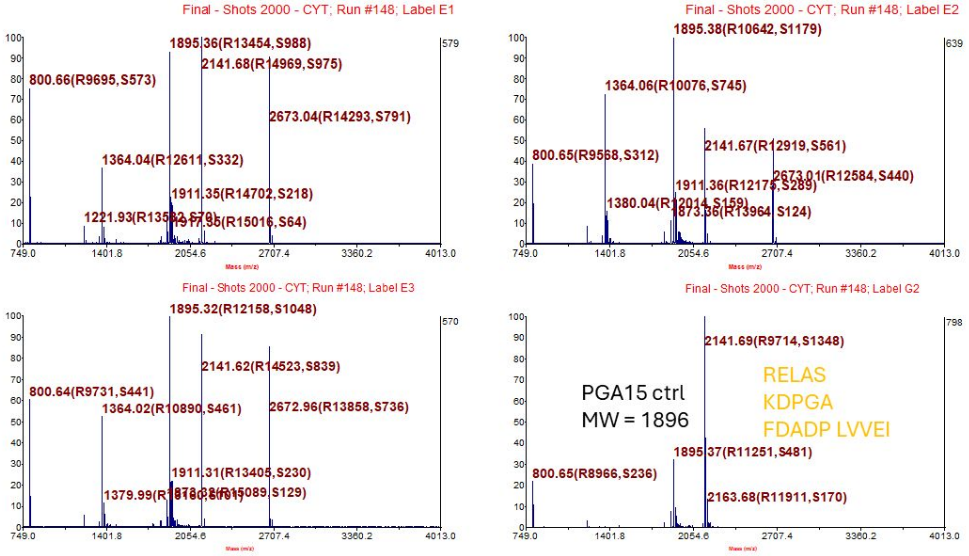

AGA15 5KDPGA10

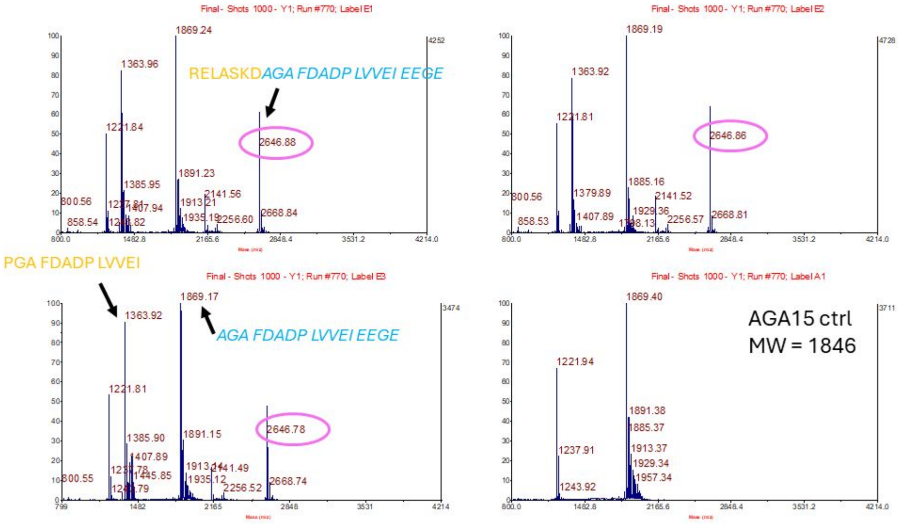

RGA15 5KDPGA10

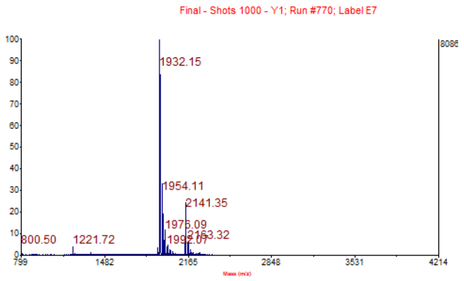

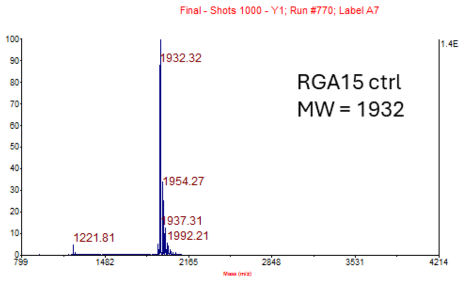

RGA15, no ligation with 5KDPGA10

DGA15 5KDPGA10

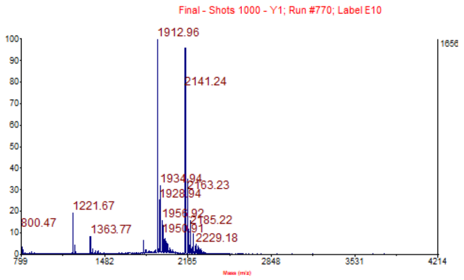

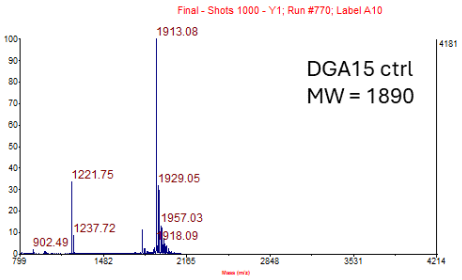

DGA15, no ligation with 5KDPGA10

VGA15 5KDPGA10

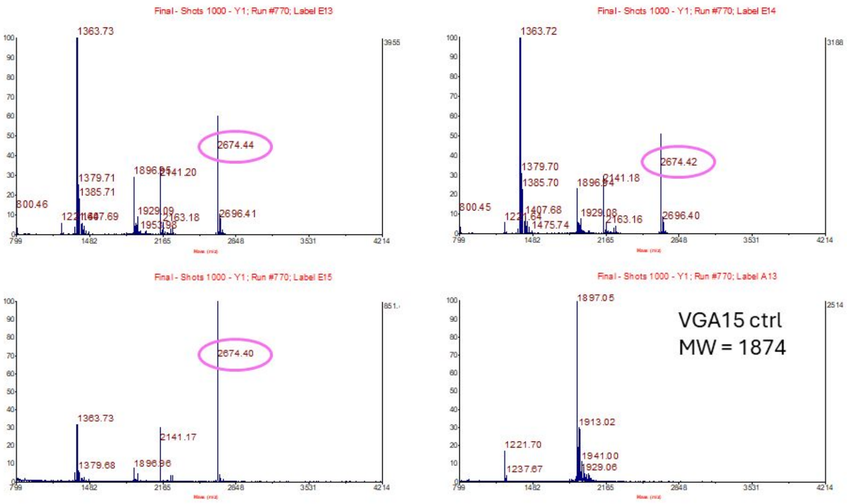

YGA15 5KDPGA10

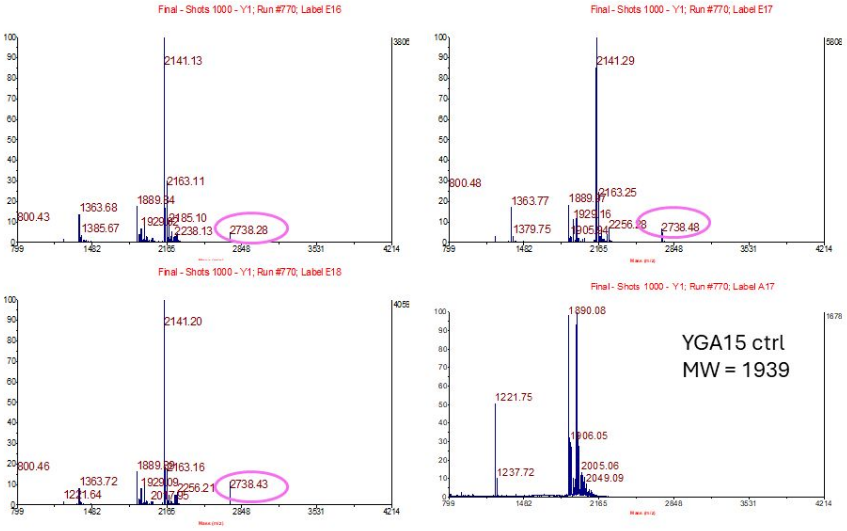

YGA15 stock sln was degraded (no peak of target MW1939)

LGA15 5KDPGA10

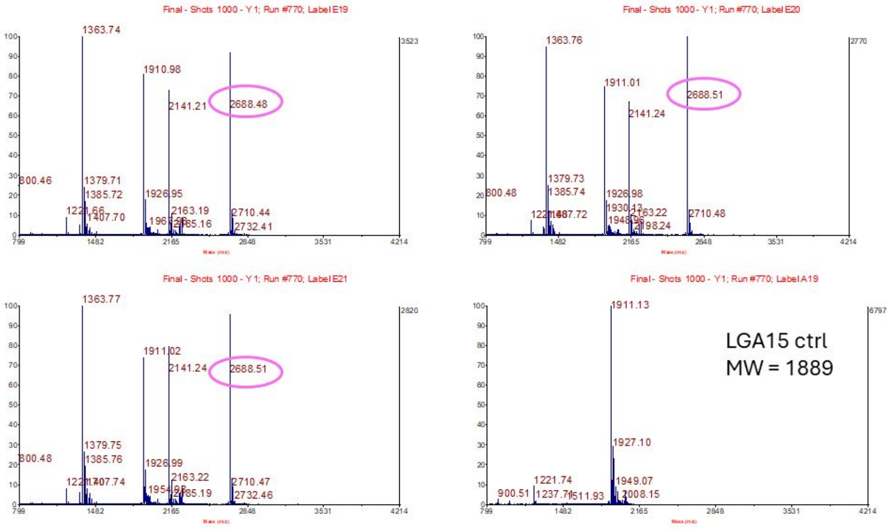

NGA15 5KDPGA10

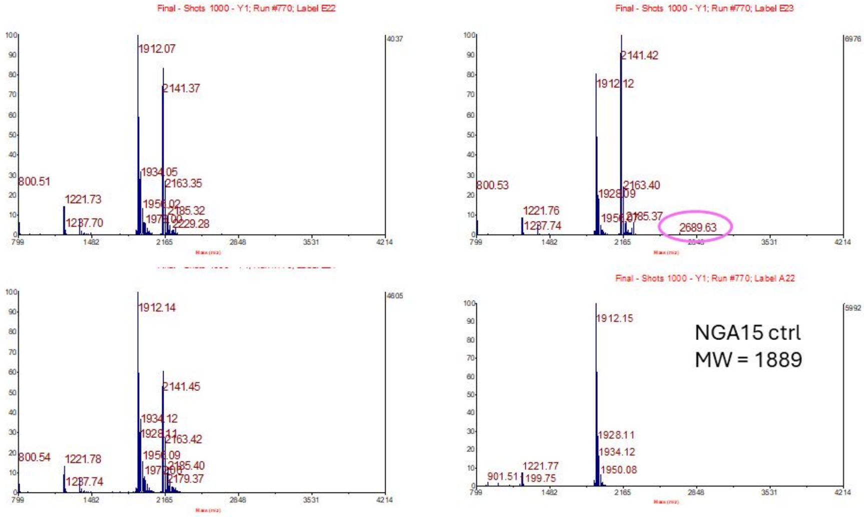

CGA15 5KDPGA10

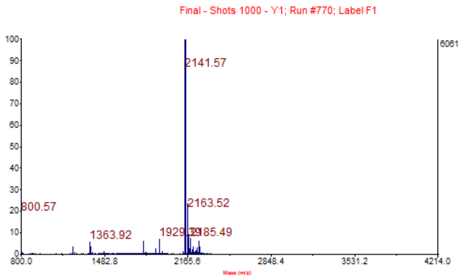

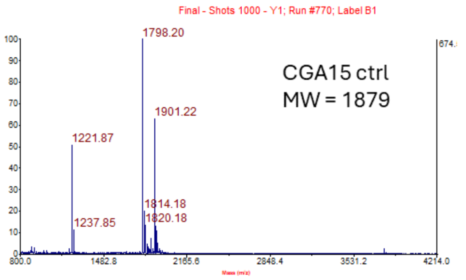

CGA15, no ligation with 5KDPGA10

KGA15 5KDPGA10

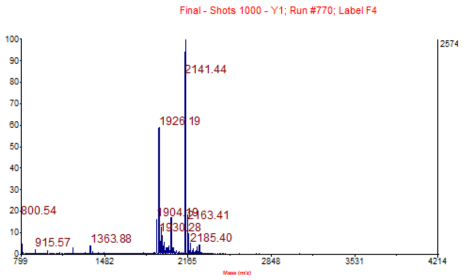

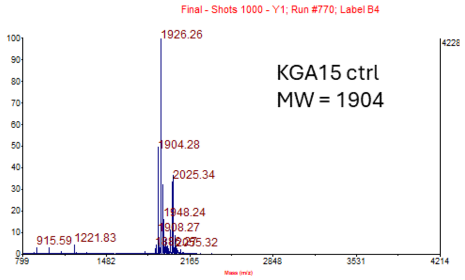

KGA15, no ligation with 5KDPGA10

MGA15 5KDPGA10

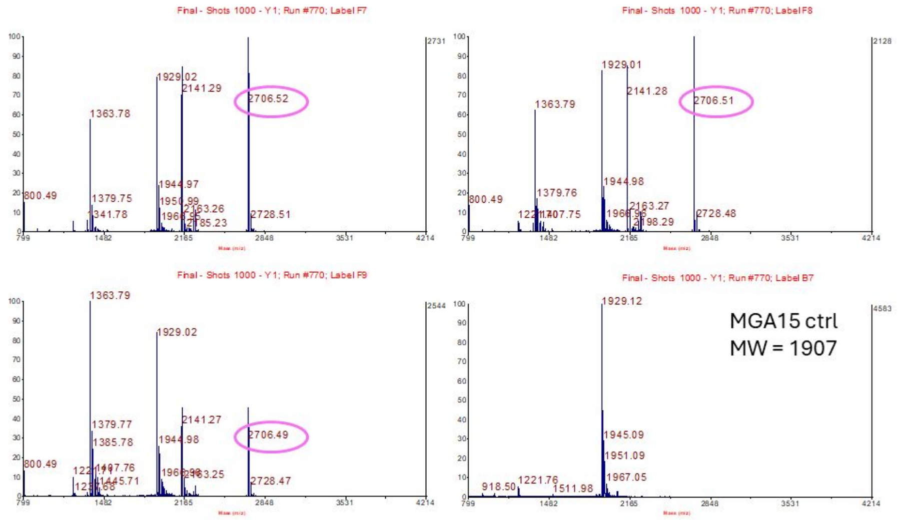

HGA15 5KDPGA10

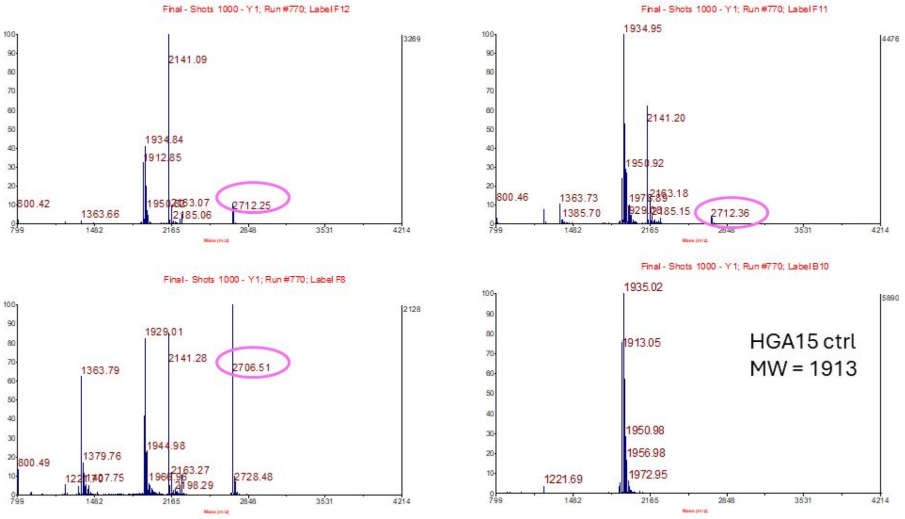

SGA15 5KDPGA10

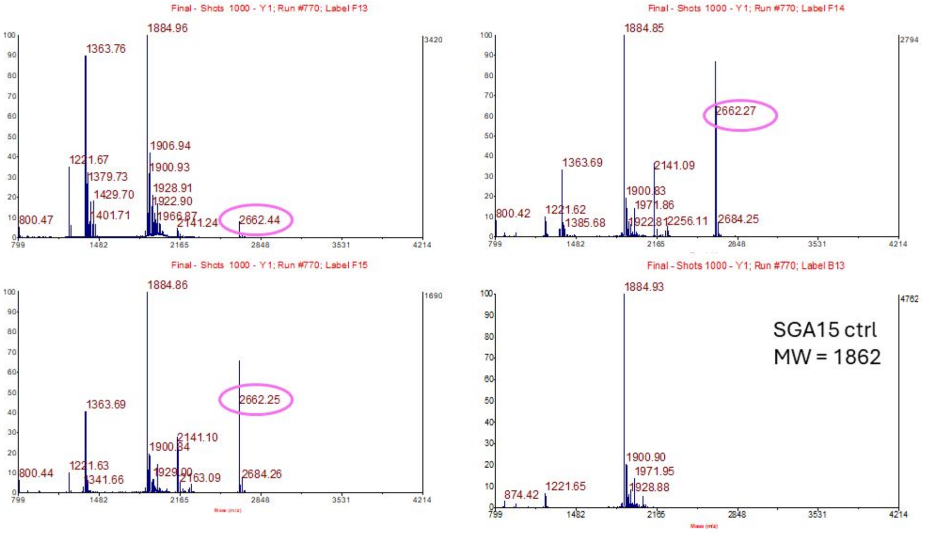

FGA15 5KDPGA10

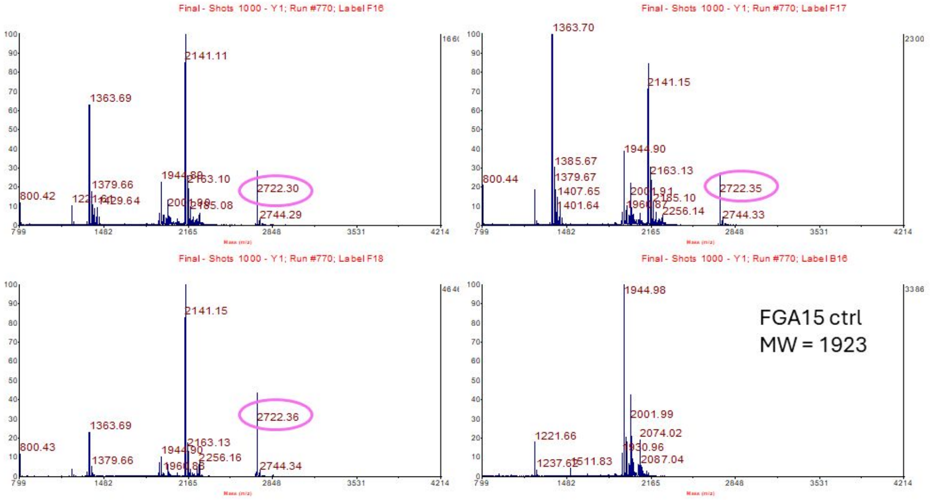

EGA15 5KDPGA10

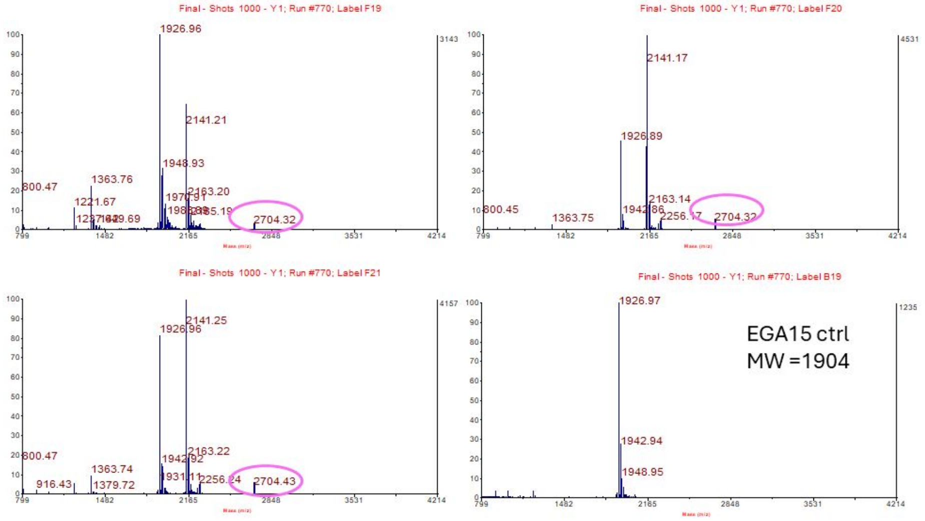

QGA15 5KDPGA10

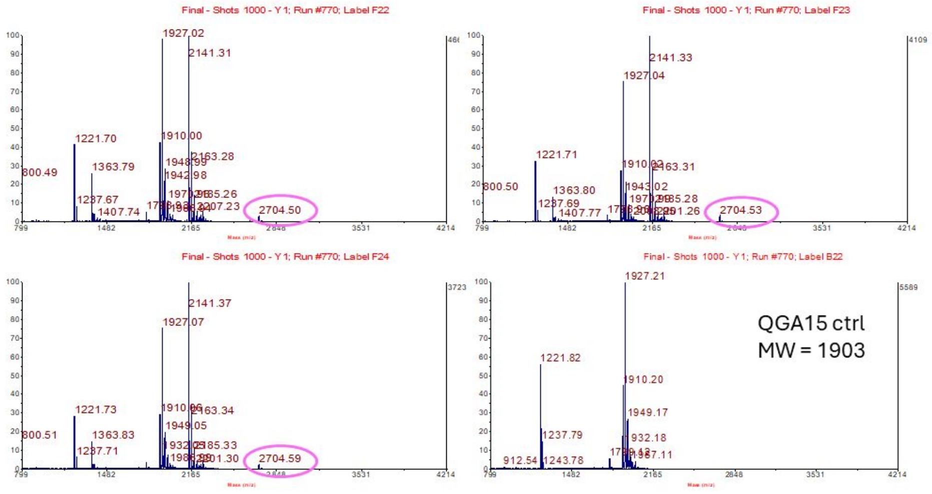

GGA15 5KDPGA10

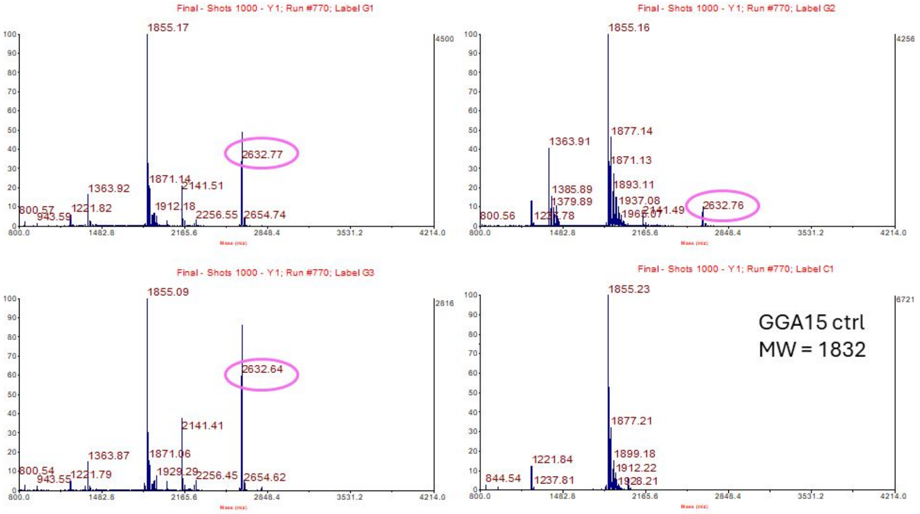

IGA15 5KDPGA10

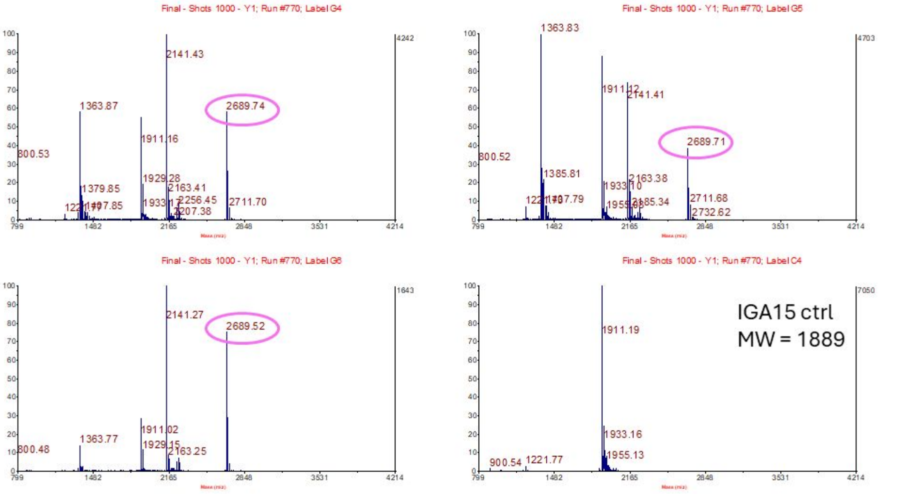

WGA15 5KDPGA10

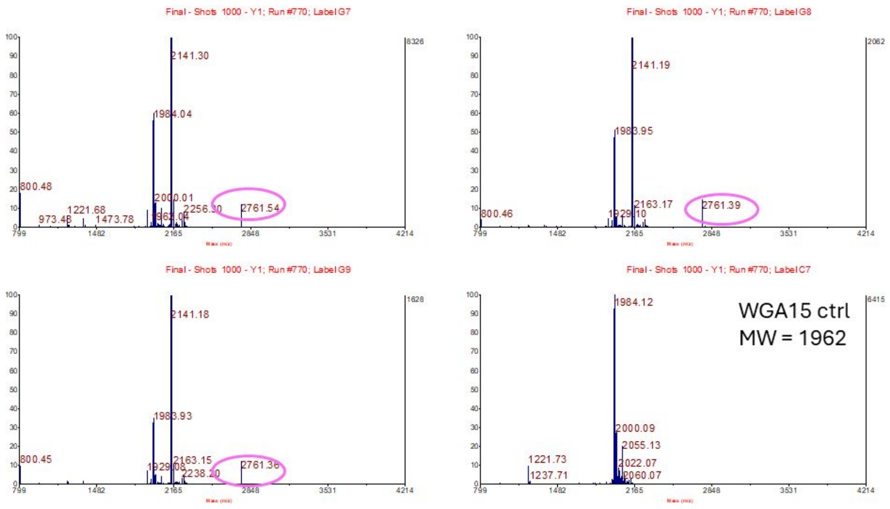

TGA15 5KDPGA10

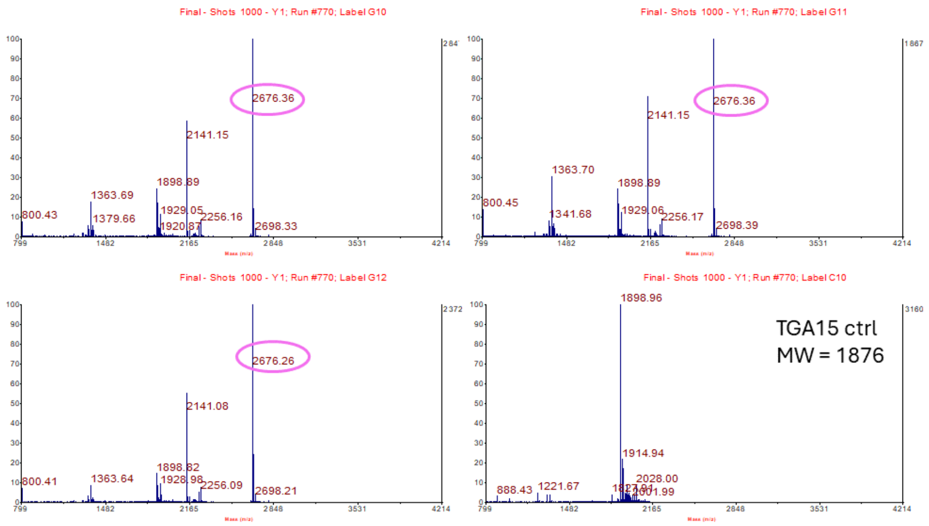

Supplementary figure 7. Mass-spec spectrum of XGA N-terminal substrate and product peptides

15 min PGA FDADP LVVEI SEEGE + RELAS KDPGA FDADP LVVEI

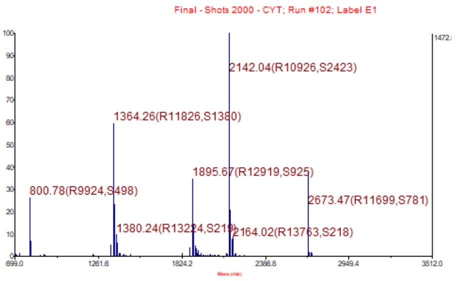

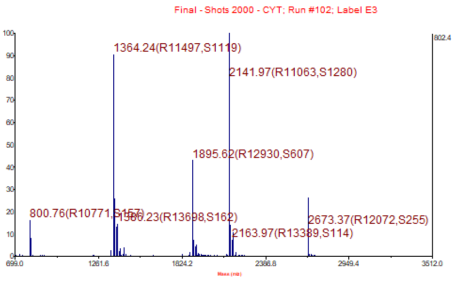

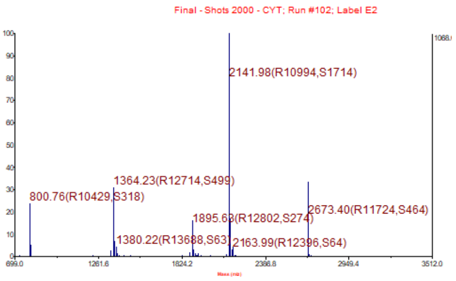

30 min PGA FDADP LVVEI SEEGE + RELAS KDPGA FDADP LVVEI

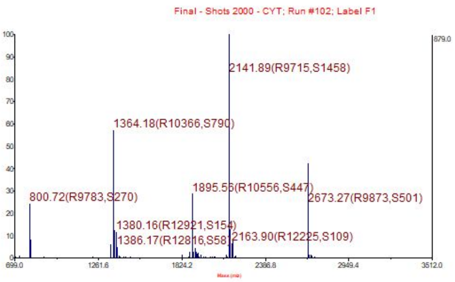

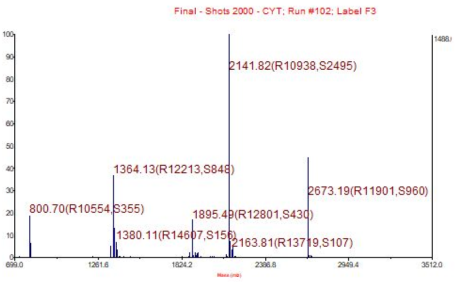

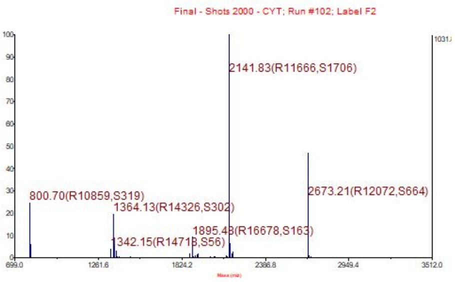

90 min

PGA FDADP LVVEI SEEGE + RELAS KDPGA FDADP LVVEI

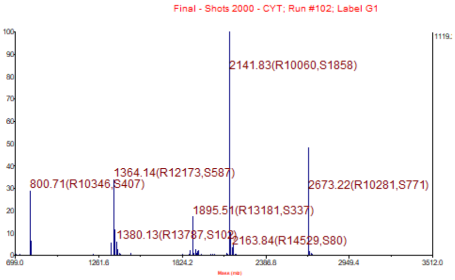

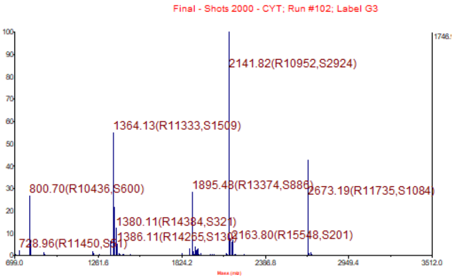

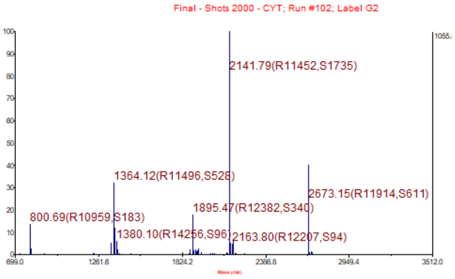

15 min

GGA FDADP LVVEI SEEGE + RELAS KDPGA FDADP LVVEI

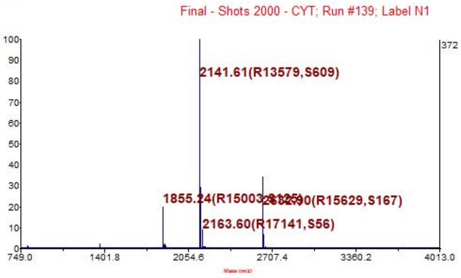

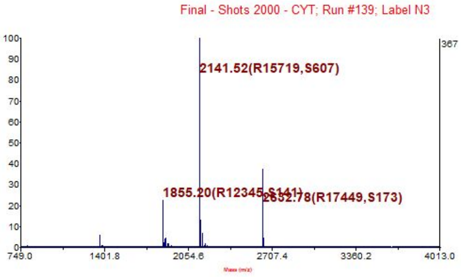

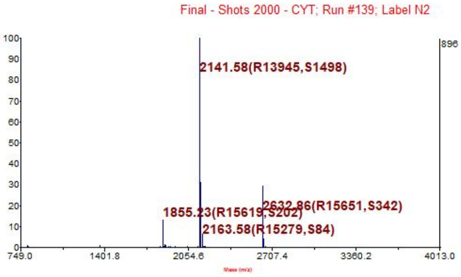

30 min GGA FDADP LVVEI SEEGE + RELAS KDPGA FDADP LVVEI

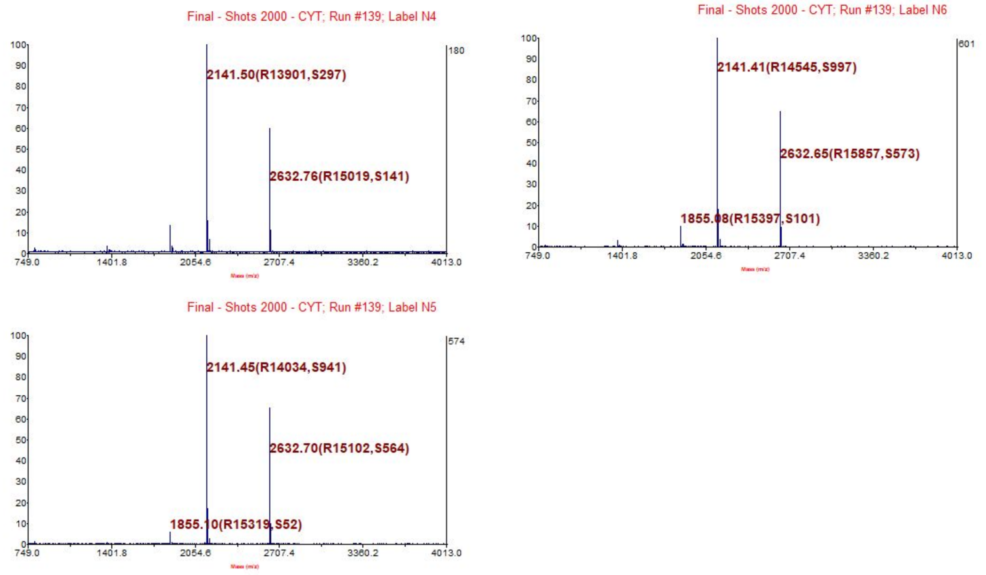

90 min GGA FDADP LVVEI SEEGE + RELAS KDPGA FDADP LVVEI

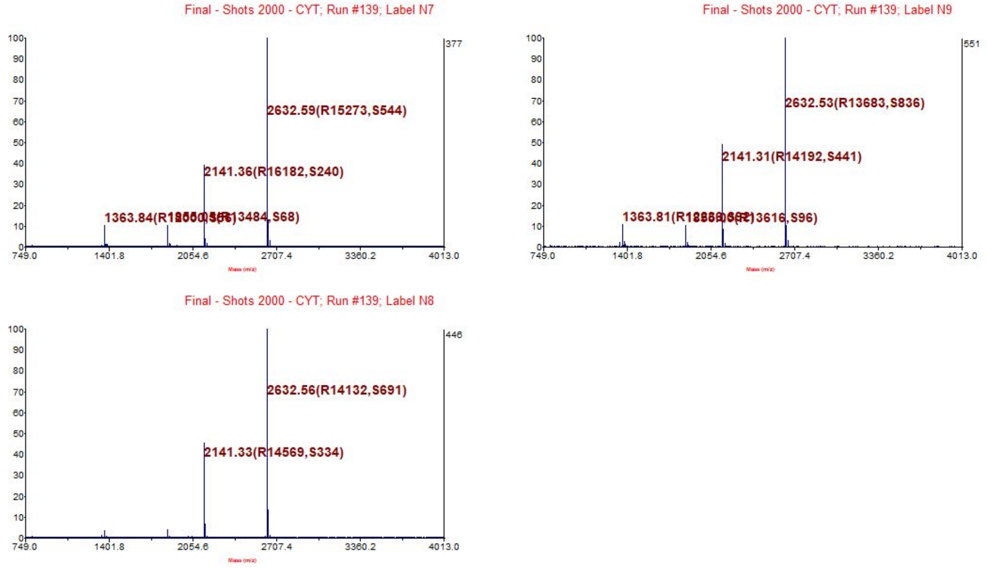

Supplementary figure 8. Mass-spec spectrums of time-based PGA/GGA substrate and product peptides

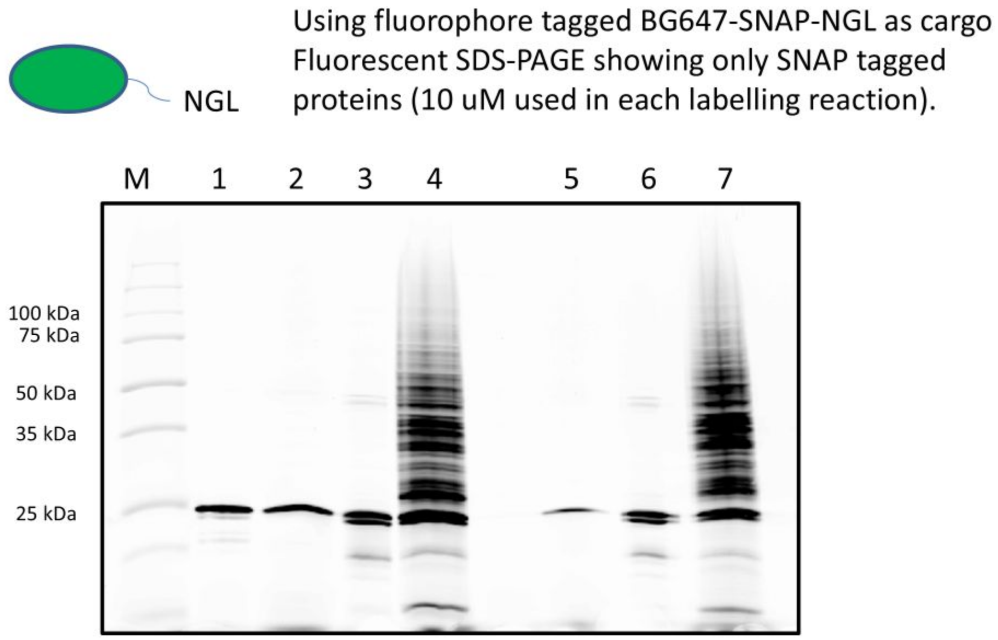

Supplementary figure 9. Non-specific cellular surface labelling conducted by 1μM OaAEP1 (C247A). Lane M is molecular marker indicated for standard molecular weight. Lane 1 is SNAP-NGL protein control (10 μM). Lane 2 is SNAP-NGL added to RAW cells, no OaAEP1 (C247A), then harvested directly. Lane 3 is SNAP-NGL added to RAW cells, with OaAEP1 (C247A), 1X wash solution after 20 minutes incubation. Lane 4 is SNAP-NGL added to RAW cells, with OaAEP1 (C247A), cells harvested after 3X wash, then resuspended and lysed for SDS-PAGE analysis. All SNAP protein tags were prelabeled with BG-647, and quenched with 100X excess BG. Lane 5-7 are the same samples except replacing RAW cells with HEK293T cells.

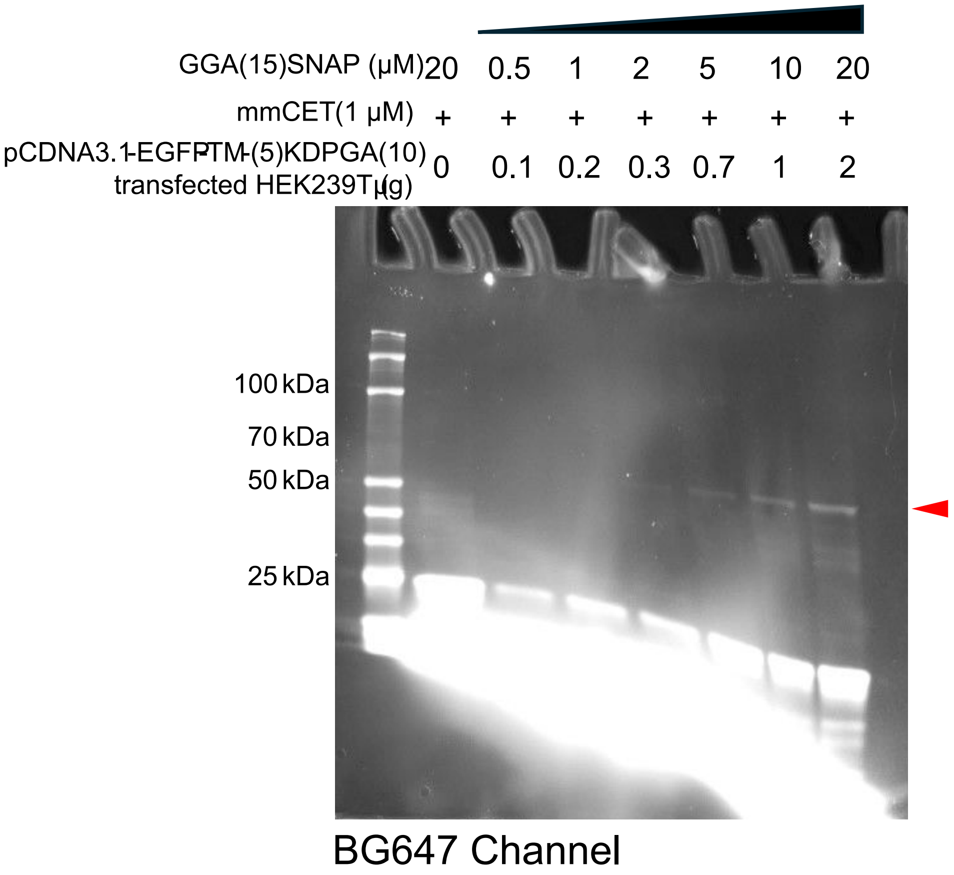

Supplementary figure 10. Cellular surface labelling on HEK293T cells by Connectase (mmCET). HEK293T cells were transfected with increase plasmid concentration of pCDNA3.1 EGFP-TM-(5)KDPGA(10) to express the surface C-terminal motif. Post transfected cells were incubated with various titrations of GGA(15)-SNAP with connectase (mmCET) in their culturing media for 15min at 37°C. Harvested cells were resuspended and lysed for SDS-PAGE analysis. All SNAP protein tags were prelabeled with BG-647. Fluorescence detection of BG647 for SDS-PAGE gels run of cellular lysates The red arrows denoted the ligated cargo onto the anchor protein on the cellular surface.

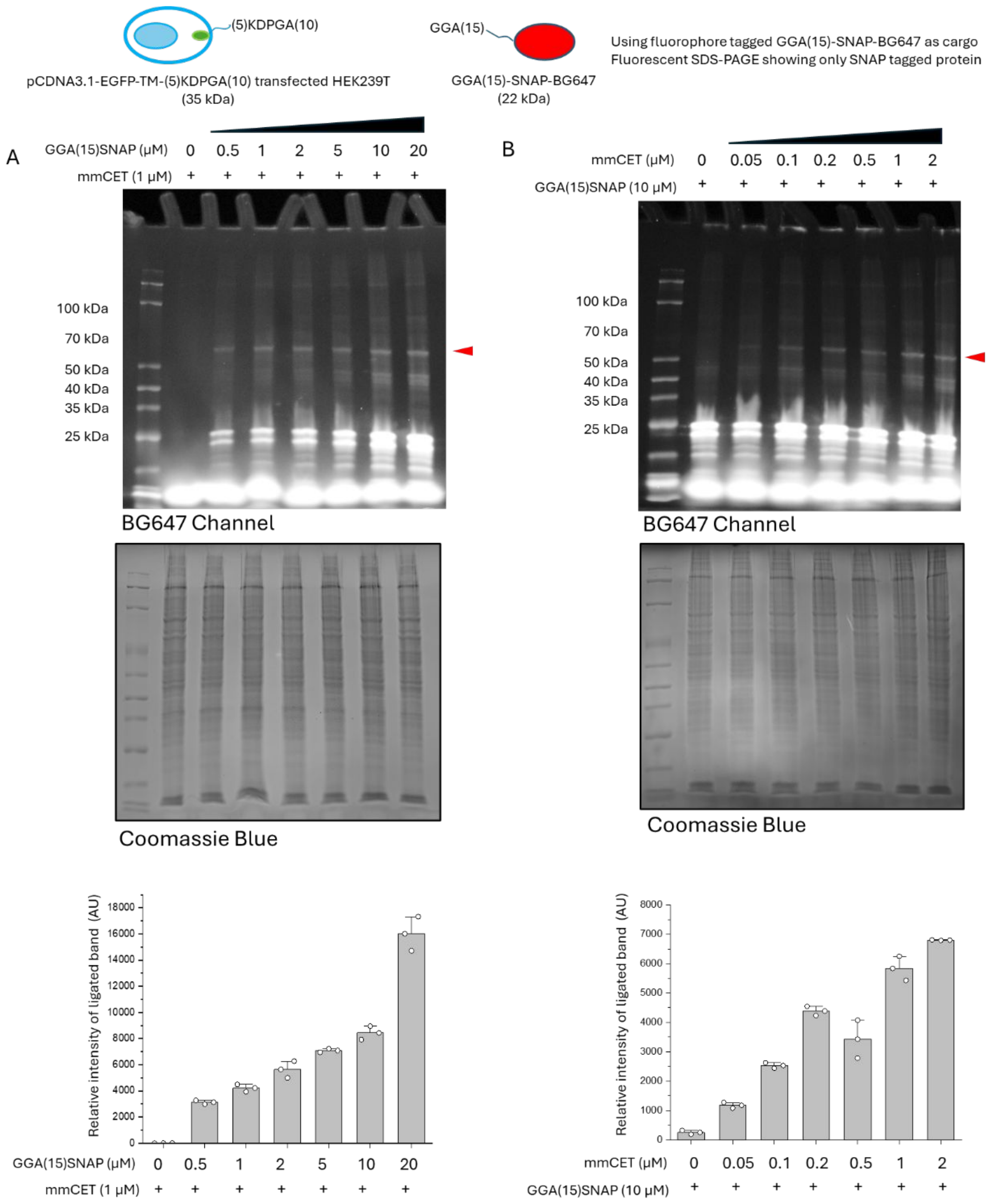

Supplementary figure 11. Cellular surface labelling on HEK293T cells by Connectase (mmCET). Fluorescence detection of BG647 for both SDS-PAGE gels run of cellular lysates. HEK293T cells were transfected with pCDNA3.1 EGFP-TM-(5)KDPGA(10) to express the surface C-terminal motif. Post transfected cells were incubated with (A) various titrations of GGA(15)-SNAP with connectase (mmCET) and (B) various titrations of mmCET in the presence of 10μM GGA(15)-SNAP in their culturing media for 15min at 37°C. Harvested cells were resuspended and lysed for SDS-PAGE analysis. All SNAP protein tags were prelabeled with BG-647. The graph plots the relative intensity of the ligated band from the BG647 channel normalized to Coomassie blue band using imageJ software. The red arrows denoted the ligated cargo onto the anchor protein on the cellular surface.

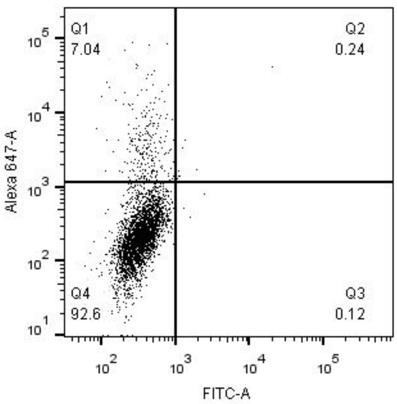

Untransfected HEK239T + mmCET

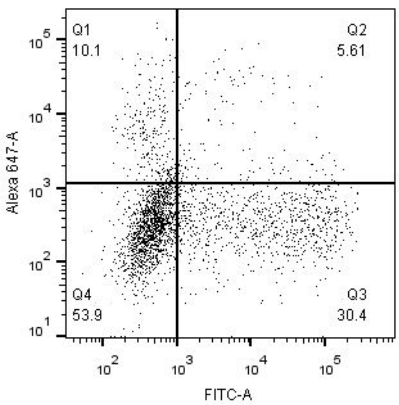

EGFP-TM-(5)KDPGA(10) HEK239T +
10uM N-ter recognition motif FLAG

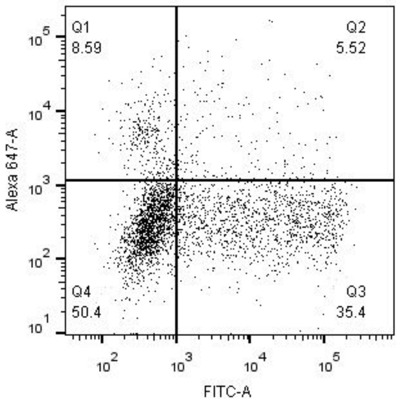

EGFP-TM-NAL HEK239T +

10uM N-ter recognition motif FLAG

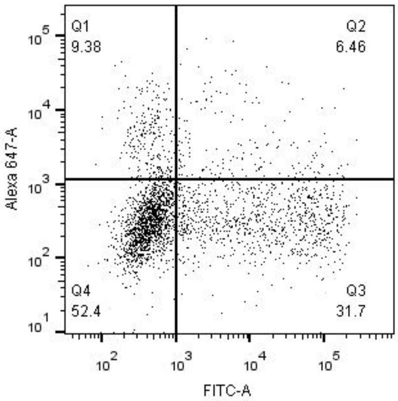

EGFP-TM-NAL HEK239T +

10uM N-ter recognition motif FLAG +

1uM mmCET

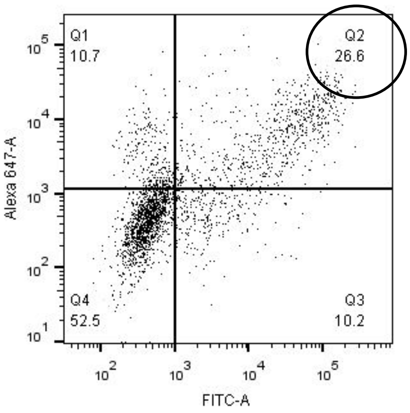

EGFP-TM-(5)KDPGA(10) HEK239T +
10uM N-ter recognition motif FLAG +

1uM mmCET

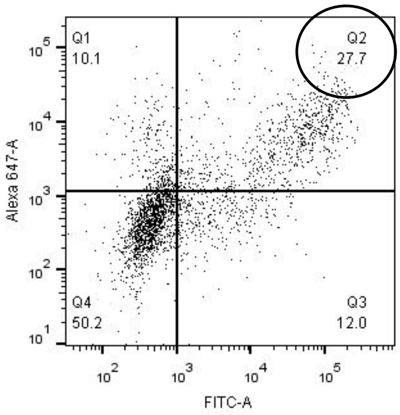

EGFP-TM-(5)KDPGA(10) HEK239T +
10uM N-ter recognition motif FLAG +

5uM mmCET

Supplementary figure 12. Fluorescence imaging and flow cytometry analysis to validate cellular surface ligation of Connectase (mmCET) in HEK293T cells, under cell culture condition. Flow cytometry analysis of cellular surface ligation of Connectase (mmCET) in HEK293T cells.

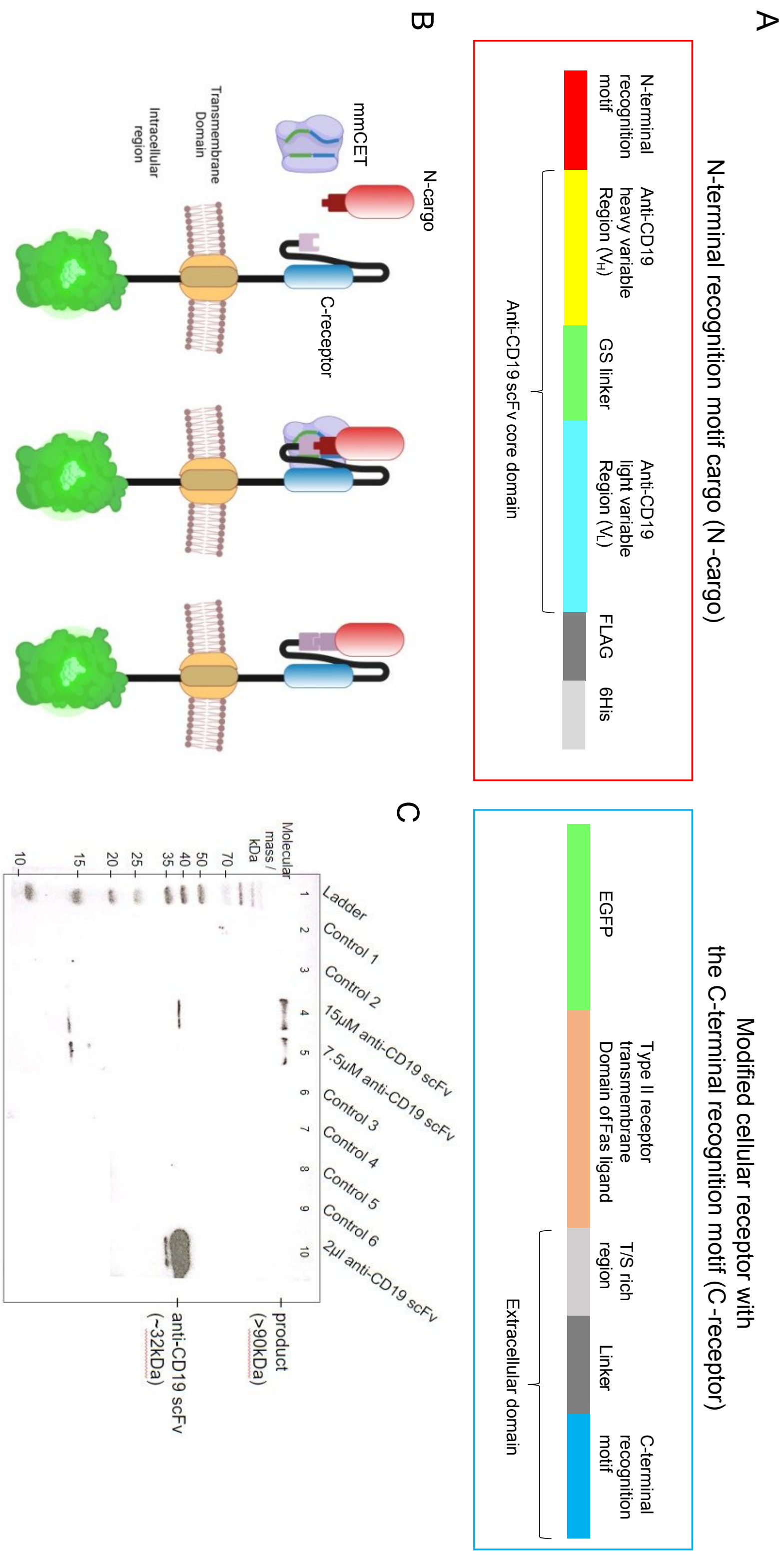

Supplementary figure 13. Target protein ligation on cellular surface. A) Construct of N-terminal recognition motif cargo and modified cellular receptor with the C-terminal recognition motif. B) Diagram showing N-cargo being ligated to C-receptor with the presence of mmCET on a cellular surface. C) Immunoblot detected with anti FLAG probe showing cellular surface ligation with target protein.

Table S1. X-ray Crystallography data collection and refinement statistics

	mmCnt^{T1A}	***mmCnt***$^{T1A/C192S}$ ***with substrate***
**Crystal parameter**		
Space group	P 31 2 1	P 21 21 2
Cell dimensions		
a, *b*, *c* (Å)	90.72, 90.72, 90.46	54.10, 100.97, 32.60
α, β, γ (°)	90, 90, 120	90, 90, 90
Subunit	2	2
**Data collection**		
Beamline	Aus Sync MX1	Aus Sync MX1
Wavelength (Å)	1.0	0.9537
Resolution (Å) [a]	45.36 – 3.40 (3.68 – 3.4)	47.69 – 1.98 (2.07 – 1.98)
No. observations	25516	61160
R_{merge} [a]	0.14 (1.32)	0.19 (1.52)
$I / \sigma I$ [a, b]	13.2 (1.7)	9.2 (1.4)
Completeness (%) [a]	99.7 (98.6)	99.4 (94.7)
Redundancy [a]	9.9 (10.2)	12.5 (10.6)
**Refinement**		
Resolution (Å) [a]	3.40 (3.74 – 3.4)	1.98 (2.07 – 1.98)
No. unique reflections	11443	23726
R_{work} / R_{free} [a]	0.207 / 0.285 (0.312 / 0.428)	0.231 / 0.270 (0.324 / 0.377)
Wilson B-factor (Å2)	118.2	34.9
Anisotropy	0.468	0.337
Bulk solvent *ksol*(e/Å3), *Bsol*(Å2)	0.27, 161.9	0.34, 37.4
Total number of atoms	2944	1664
B-factors	226.0	46
R.m.s. deviations [c]		
Bond lengths (Å)	0.019	0.011
Bond angles (°)	2.42	1.53
Ramachandran Plot (%) [d]	97.56/1.95/0.49	96.59/1.95/1.46
PDB accession code	8JTU	8WKD

[a] The values in parentheses correspond to the highest resolution shell.

[b] Intensities estimated from amplitudes.

[c] Deviations from ideal bond lengths/angles.

[d] Percentage of residues in favored region/allowed region/outlier region.

EXPERIMENTAL METHODS

Plasmid construction. To generate expression plasmids for Connectase wildtype (residues 1-193), nucleotide sequence was obtained from NCBI search (Accession #: AAM32605.1) and synthesized by Biobasic gene synthesis platform in a pET47b(+) backbone containing C' terminal hexa-His tag for purification. To generate Connectase mutants, site-directed mutagenesis was performed using the Q5® High-Fidelity PCR kits (New England Biolabs, SG) and specific base substitution primers. mCherry, SNAP and Ubiquitin ligation substrates for protein-protein ligation assays were cloned into pET47b(+) plasmid backbones with double restriction digestion and T4 ligation, followed by site-directed mutagenesis to insert N' terminal Connectase recognition sequence (PGA FDADP LVVEI) and C' terminal Connectase recognition sequence (RELAS KDPGA FDADP LVVEI) respectively. Nucleotides and protein sequences are listed in Table S2.

Protein expression and purification. To obtain protein substrates, plasmids constructed were transformed into BL21(DE3) (NEB, C2527) and grown in Miller's LB broth (LB, Biobasic) media at 37°C for 16–18 h, in a shaking incubator (220rpm). Starting culture was then used to inoculate a larger volume of lysogeny broth, which was grown to OD600 of ~0.6–0.8. Temperature was then lowered to 16 °C, protein expression was induced with 0.5 mM IPTG and bacteria were cultured overnight. Bacteria were harvested at 4000 ×g (Avanti JXN series, Beckman Coulter) for 15 minutes and then resuspended with lysis buffer (50 mM Tris-HCl, pH 7.4, 150 mM NaCl, 0.05% (v/v) CHAPS, 10 % (v/v) glycerol. Cells were then lysed by Emulsiflex-C3 Homogenizer and centrifuged at a speed of 30,000 × g (Avanti JXN series, Beckman Coulter) for 30 minutes at 4°C. The supernatant was passed through a pre-equilibrated column containing Ni-NTA agarose beads (BioBasic, SA005100) by gravity. The column was then washed with lysis buffer (50 mM Tris-HCl, pH 8.0, 150 mM NaCl, 0.05% (v/v) CHAPS, 10 % (v/v) glycerol) containing 20 mM imidazole to remove any non-specific binding proteins. His-tagged proteins were then eluted with increasing imidazole concentration (100mM-300mM, each step a 50mM increment) and verified via SDS-PAGE. To analyse the eluted fractions, Sodium Dodecyl-Sulfate Polyacrylamide Gel Electrophoresis (SDS-PAGE) was carried out. Fractions containing the protein were then concentrated using Amicon Ultra concentrator (Merck Millipore, USA) and further purified on a Size Exclusion column (Superdex™ 200 10/300 GL, GE Healthcare, USA) in elution buffer (20 mM Tris and 100 mM NaCl, pH 8.0).

Crystallization of Connectase T1A, crystallographic data collection and structure determination. Protein at a concentration of 30 mg/ml was subjected to robotic crystallization trials using the sitting drop/vapor diffusion method and a Mosquitoe instrument (TTP Biotech UK). Two protein concentration (15 mg/mL and 30 mg/mL) and an absence of protein were tested with commercial crystallization screens (Molecular Dimensions, Hampton Research) in intelli 96-3 wells sitting drop plates. Optimized Connectase T1A crystals were by mixing equal volume of protein and a precipitate solution containing 0.1 M Tris-HCl, pH 8.5, 150 mM NaCl, 12% PEG 20k. Crystals were cryo- protected by a brief soak in the precipitating solution supplemented with 10% (v/v) glycerol and rapidly frozen in liquid nitrogen. The diffraction intensities of crystal were collected at MX1 beamline, Australian Synchrotron (Melbourne, Australia) to a resolution of 3.15 Å. A total of 720 images of 0.25° oscillation each, was processed with the CCP4 package. Diffraction intensities were

integrated with program Imosflm and scaled with program SCALA from the CCP4 suite. The crystal structure of Connectase T1A was determined by molecular replacement using M.J connectase as a search probe (PDB codes 6ZW0). Model rebuilding sessions at the computer graphics, using program Coot were interspersed with refinement to a resolution of 2.0 Å using Phenix. Data collection statistics are summarized in Table S1. Figure illustrations were produced using Pymol. The atomic coordinates and structure factors are deposited with the Protein Data Bank under accession code 8JTU and 8WKD.

Protein-Protein Ligation Assay. Protein-protein ligation assay involves ligating two proteins using Connectase. A standard protein-protein ligation reaction consists of two substrates e.g., PGA(10) SNAP and Ubi (5)KDPGA(10), and Connectase in Phosphate-Buffered Saline (PBS). The concentration used for both proteins was 10µM, whereas the concentration for Connectase varies. The reaction mix was then incubated at 37°C for 15mins. After incubation, BG-647 was added and left to sit for two mins. SDS-PAGE analysis was then performed to identify whether protein ligation is successful, by imaging the gel under BG-647 and Coomassie Blue Staining. To compare the ligation efficiency of Connectase mutants with the WT, all assay components remained the same except for the Connectase enzyme used. Different PGA(10) substrates were also tested – PGA(10) Ub FLAG, PGA(10) GSGSGS mCherry and PGA(10) Ub mCherry. Similarly, all components remained the same except for the type of PGA(10) substrate tested.

Peptides synthesis. All Fmoc amino acids powder and benzotriazol-1-yloxytripyrrolidinophosphonium hexafluorophosphate (PyBOP) were purchased from GL Biochem, Shanghai. Solvents such as dimethylformamide (DMF), N,N-Diisopropylethylamine (DIEA) and dimethylchloride (DCM) were purchased from Merck-Millipore, USA. Trifluoroacetic acid (TFA), 2,2 - (Ethylenedioxy)diethanethiol (DODT), triisopropylsilane (TIS), N,N′-Diisopropylcarbodiimide (DIC) and diethyl ether were purchased from Sigma Aldrich. OxymaPure was purchased from CEM. Piperidine was purchased from Acros. Biotin was purchased from IBA life sciences. All peptides used in this manuscript were synthesized in-house by Fmoc-based automated CEM Liberty peptide synthesizer with DIC/OxymaPure as coupling reagent and HPLC purified. All peptides identities were confirmed by MALDI TOF MS under reflective mode (ABI 4800 MALDI TOF/TOF). MBHA rink amide resin was used for solid phase synthesis with double coupling method. 20% piperidine in DMF was used as deprotection agent. Resin conjugated peptide was repeatedly washed with DMF and DCM before cleavage. A freshly prepared cleavage cocktail (94% TFA/ 2.5% H_2O/ 2.5% DODT/ 1% TIS) was added to the resin for minimum 2 hours cleavage at ambient temperature. 4 times volume of cold ether was added into the TFA peptide solution to precipitate crude peptide out from the TFA solution. The crude peptide was purified by reverse phase HPLC with BioRad NGC system, C18 analytical column (Jupiter Phenomenex 5um C18 300Å 250 x 10mm). Fractions were analysed by MALDI-TOF MS to confirm the presence of target peptide. Fractions with target peptide were lyophilised for a minimum of 24 hours. Dried peptide powder was stored at -20°C. All peptides synthesis are listed in Table S4.

Peptide-Peptide Ligation Assay. Peptide-peptide ligation assay involves ligating two peptides using Connectase. Native and modified amino acids were purchased from Sigma Aldrich (USA). All peptides used in this manuscript were synthesised by the lab using the solid phase

method with the Liberty Blue™ Automated Microwave Peptide Synthesiser (CEM Corporation, USA) and HPLC purified. The identity of each purified peptide via HPLC peak was analyzed by MALDI TOF MS (ABI 4800 MALDI TOF/TOF). A standard peptide-peptide ligation reaction consists of two peptides e.g., PGA(15) and (5)KDPGA(10), and Connectase in PBS. The concentration used for both peptides was 50μM, whereas the concentration for Connectase was 0.5μM. The reaction mix was then incubated at 37°C for 15mins. The samples were directly spotted on a Matrix-Assisted Laser Desorption/Ionization Mass Spectrometry (MALDI MS) sample plate in triplicates. α-cyano-4-hydroxycinnamic acid (CHCA), which is a recrystallized matrix for MALDI MS, was spotted on the samples. Finally, the samples were analysed using MALDI MS in linear mode, with a mass range of 820 – 5000 Da. To compare the ligation efficiency of Connectase mutants with the WT, all assay components remained the same except for the Connectase enzyme used.

Glass Surface Functionalization and Protein Immobilization. AFM probes (MLCT-BIO-DC, Bruker Corp.) and glass coverslips were placed in a plasma cleaner for 5 minutes and 25 minutes, respectively. They were then amino-functionalized by immersing in 1.5% (v/v) 3-aminopropyltriethoxysilane (APTES) in toluene for an hour. A solution mixture of 4 mM ImSO2N3, 8 mM K2CO3, and 40 μM CuSO4 was used to convert the amino groups into azide groups. A 2 mg/mL concentration of C-terminal alkynyl-modified peptide in Tris-HCl buffer (100 mM Tris, 100 mM NaCl, pH 7.4) was reacted with the azide group on the coverslip through Cu-catalyzed azide-alkyne cycloaddition (CuAAC) click reaction. The AFM tip was functionalized with DBCO-PEG4-maleimide (4 mM in DMSO), Cys-ELP20-NGL, and GL-GB1-XDoc as reported. coverslips and tips were dried by gently blowing N2 and kept in a 4°C refrigerator. A 40 μL protein solution of Coh-(5)KDPGA(10) (~100 μM) in Tris-HCl buffer was added to the functionalized substrate with 5 μL connectase (50 μM). After incubation at room temperature for an hour, the surface was washed with Tris buffer. Additionally, GL-Ub-(5)KDPGA(10) (40 μL, 500 μM) was ligated between the substrate and Coh-NGL at the OaAEP1 ligation site NGL and the connectase ligation site (5)KDGGA(10). For further study, GL-Ub-(5)KDPGA(10) (40 μL, 500 μM) and GGA(10)-I27-NGL (40 μL, 500 μM) units were linked into polyproteins by sequential use of OaAEP1 and connectase. Tris buffer was thoroughly used to wash away enzymes and unreacted units after each step. Before AFM measurement, the capping protein Coh-NGL or Coh-(5)KDPGA(10) (~200 μM) was added and reacted last.

Single-Molecule AFM Experiments. The AFM-SMFS experiments were carried out on a commercial AFM (JPK ForceRobot 300) at room temperature. The tip was retracted at a speed of 400 nm/s. The captured polyproteins were stretched and unfolded until the Coh-XDoc interaction ruptured. The force spectroscopy data were first filtered and analyzed using data processing software (JPK). Curves that showed strong rupture forces over 350 pN were selected for analysis.

Cell culture and transfection. Cell lines HEK293T was from ATCC (Manassas, VA), both cell lines were maintained at 37 °C with 5% CO2 in DMEM (Gibco) supplemented with 10% (v/v) fetal bovine serum (Gibco). Cellular viablilty checked was performed by staining the cells with Trypan Blue (ThermoFisher Scientific, SG). The stained cells were added into a Countess™ Cell Counting Chamber Slide (ThermoFisher Scientific, SG) for use with the Countess™ Automated Cell Counter (ThermoFisher Scientific, SG). HEK293T cells were plated at a density of

5 × 105 cells per well for poly-L-lysine- (Sigma-Aldrich) coated six-well plate prior to transfections. Plasmids were transfected into HEK293T cells using FuGENE HD (Promega) with a “5:1“ratio according to the manufacturer’s instructions.

Cellular Surface Ligation. The transfected cells were harvested 24 hour post-transfection by using a cell scrapper. Medium was either replaced with reaction buffer containing 0.1% BSA in 1x PBS, pH 7.4 or with fresh culturing media DMEM with 10% FBS. 1 µM of Connectase or OaAEP1 (C247A) and 10 µM of ligating substrates were added and incubated at 37°C for at least 15 mins. The cells were washed thrice with 1x PBS, pH 7.4 before further assay.

Flow cytometric assay and analysis. To the cell pellet, the following reagents were added – ice cold Fluorescence-Activated Cell Sorting (FACS) buffer containing 1X PBS and 0.5% Bovine Serum Albumin (BSA), primary mouse anti-flag M2 antibody (1:1000 dilution) (Sigma-Aldrich, SG) and secondary Alexa 647 goat anti-mouse IgG (1:250 dilution) (ThermoFisher Scientific, SG). This is followed by incubation at 4°C for 1 hour in the dark. After incubation, the cells were washed and resuspended using an ice cold FACS buffer. The cells were passed through a 5ml Polystyrene Round-Bottom Tube with Cell-Strainer Cap, to obtain single cells. Finally, the cells were analyzed using BD LSRFortessa™ X-20 Cell Analyzer and fcs files is processed using FlowJo Version 10 (FlowJo, LLC).

Immunoblotting. Cells were lysed with ice-cold RIPA lysis buffer (20 mM $Na_2H_2PO_4$, 250 mM NaCl, 1% Triton X-100, 0.1% SDS, pH 8.0). Total protein lysates were resolved using 15% SDS-PAGE and electro-transferred onto Immun-Blot® polyvinylidene fluoride (PVDF) membrane (Bio-Rad, USA). Membranes were blocked with 5% Blotting-Grade Blocker (#1706404, Bio-Rad, USA) diluted with TBST (50 mM Tris HCl, pH 7.6, 150 mM NaCl, and 0.05% Tween-20) for 1 h at room temperature. The membrane was then incubated overnight at 4°C with the indicated primary antibodies in 5% BSA diluted with TBST. Membranes were washed thrice with TBST, and incubated with appropriate HRP-conjugated anti-IgG secondary antibodies (Santa Cruz Biotehnology, USA) for 1 h at room temperature. Protein bands were revealed using ECL™ Prime Western Blotting System (RPN2232, GE Healthcare Bio-Sciences, USA) and imaged using CCD-based ChemiDoc™ Imaging Systems (Bio-Rad, USA).

Table S2. Nucleotide and Protein sequence list

Nucleotides Sequence	
m. mazei **Connectase**	**Sequence (5' - 3')**
T1A	GGAGATATACATATG GCACTGGTTATCGCGTTCATCGG
E34A	GATCGTGAAAAACTGGCAAAAGAACTGTACAGCGGCAGCATC
Y38A	CTGGAAAAAGAACTGGCAAGCGGCAGCATCGTTACCGATGAAG
Y38D	GAAAAACTGGAAAAAGAACTGGACAGCGGCAGCATCGTTACCG
V79A	GTTCTGGTTGGCGAAGCAAGCAGCGCGGAAGGC
S81A	
S81G	GTTGGCGAAGTTAGCGGAGCGGAAGGCGGCGTTG
S81D	GTTGGCGAAGTTAGCGACGCGGAAGGCGGCGTTG
E83A	GTTGGCGAAGTTAGCAGCGCGGCAGGCGGCGTTGTTAAAAAG
N125S	CTTCATCGCGTTCGGT AGC GAATTCACCAAACAGGTTGCG
N125A	CTTCATCGCGTTCGGT GCG GAATTCACCAAACAGGTTGCG
N125G	CTTCATCGCGTTCGGT GGC GAATTCACCAAACAGGTTGCG
**Ubiquitin**	**Sequence (5' – 3')**
(5)KDPGA(10)	CCTGCGTCTGCGCGGTGGTAATGGTCTTAGAGAGCTAGCAAGCAAGGATCC<br>AGGTGCTTTCGACGCAGATCCACTAGTAGTCGAAATATGAGGATCCTAACTC<br>GAGGC
**SNAP**	**Sequence (5' – 3')**
PGA(10)	CCAGGTGCTTTCGACGCAGATCCACTAGTAGTCGAAATA
GGA(15)	GGCGGTGCTGTTGACGCAAAGCCACTAGTAGTCGAAATACCCGTGGATATC<br>AAG
**mCherry**	**Sequence (5' – 3')**
PGA(10)Ub	GTCGAAATACCCGTGGATATCAAGATGCAGATCTTCGTGAAAACCCTGACCG<br>GCAAGACCATCACCCTCGAGGTGGAGCCCAGTGACACCATCGAGAATGTCA<br>AGGCAAAGATCCAAGATAAGGAAGGCATCCCTCCTGATCAGCAGAGGTTGA<br>TCTTTGCTGGGAAACAGCTGGAAGATGGACGCACCCTGTCTGACTACAACAT<br>CCAGAAAGAGTCCACTCTGCACTTGGTCCTGCGTCTGCGCCTTATCGGTGAA<br>TTCGCTGGCTCAGGATCCGGATCAGTGAGCAAGGGC
Protein Sequence	
Ubiquitin-*Oa*AEP1(C247A)	MGMAHHHHHHMQIFVKTLTGKTITLEVEPSDTIENVKAKIQDKEGIPPDQQRLI<br>FAGKQLEDGRTLSDYNIQKESTLHLVLRLRGGARDGDYLHLPSEVSRFFRPQETN<br>DDHGEDSVGTRWAVLIAGSKGYANYRHQAGVCHAYQILKRGGLKDENIVVFM<br>YDDIAYNESNPRPGVIINSPHGSDVYAGVPKDYTGEEVNAKNFLAAILGNKSAIT<br>GGSGKVVDSGPNDHIFIYYTDHGAAGVIGMPSKPYLYADELNDALKKKHASGTY<br>KSLVFYLEACESGSMFEGILPEDLNIYALTSTNTTESSWAYYCPAQENPPPPEYNV<br>CLGDLFSVAWLEDSDVQNSWYETLNQQYHHVDKRISHASHATQYGNLKLGEE<br>GLFVYMGSNPANDNYTSLDGNALTPSSIVVNQRDADLLHLWEKFRKAPEGSAR<br>KEEAQTQIFKAMSHRVHIDSSIKLIGKLLFGIEKCTEILNAVRPAGQPLVDDWACL<br>RSLVGTFETHCGSLSEYGMRHTRTIANICNAGISEEQMAEAASQACASIP

GL-Ub-(5)KDPGA(10)	MGLGSGSASGSQIFVKTLTGKTITLEVEPSDTIENVKAKIQDKEGIPPDQQRLIFAGKQLEDGRTLSDYNIQKESTLHLVLRLRAARS(KELAS)KDPGA(FDADPLVVEI)HHHHHH
GGA(10)-I27-NGL	MGGA(FDADPLVVEI)GSLIEVEKPLYGVEVFVGETAHFEIELSEPDVHGQWKLKGQPLAASPDCEIIEDGKKHILILHNCQLGMTGEVSFQAANTKSAANLKVKELAARSNGLHHHHHH
C-terminal alkynyl-modified peptide	GGA(FDADPLVVEI)GSGSGS{PRA}

Table S3. Plasmid sequences

pET47b(+)_*m. mazei*_Connectase_6His

MTLVIAFIGKNGAVMAGDMREITFEGEKPDREKLEKELYSGSIVTDEEMQKKAEEFGVKITVADCKEKVS
ERNGVLVGEVSSAEGGVVKKRRLYASAGNFAIAELINTEMTLTSQGKGSNFIAFGNEFTKQVANKCFKDN
WTKKSNLQDAVKILILCMETVARKTASVSKQFMIVQTASNADVLKVVEKDRNCGSHHHHHH*

pET47b(+)_*m. mazei*_Connectase T1A_6His

MALVIAFIGKNGAVMAGDMREITFEGEKPDREKLEKELYSGSIVTDEEMQKKAEEFGVKITVADCKEKVS
ERNGVLVGEVSSAEGGVVKKRRLYASAGNFAIAELINTEMTLTSQGKGSNFIAFGNEFTKQVANKCFKDN
WTKKSNLQDAVKILILCMETVARKTASVSKQFMIVQTASNADVLKVVEKDRNCGSHHHHHH*

pET47b(+)_*m. mazei*_Connectase_E34A_6His

MTLVIAFIGKNGAVMAGDMREITFEGEKPDREKLAKELYSGSIVTDEEMQKKAEEFGVKITVADCKEKVS
ERNGVLVGEVSSAEGGVVKKRRLYASAGNFAIAELINTEMTLTSQGKGSNFIAFGNEFTKQVANKCFKDN
WTKKSNLQDAVKILILCMETVARKTASVSKQFMIVQTASNADVLKVVEKDRNCGSHHHHHH*

pET47b(+)_*m. mazei*_Connectase_Y38A_6His

MTLVIAFIGKNGAVMAGDMREITFEGEKPDREKLEKELASGSIVTDEEMQKKAEEFGVKITVADCKEKVS
ERNGVLVGEVSSAEGGVVKKRRLYASAGNFAIAELINTEMTLTSQGKGSNFIAFGNEFTKQVANKCFKDN
WTKKSNLQDAVKILILCMETVARKTASVSKQFMIVQTASNADVLKVVEKDRNCGSHHHHHH*

pET47b(+)_*m. mazei*_Connectase_Y38D_6His

MTLVIAFIGKNGAVMAGDMREITFEGEKPDREKLEKELDSGSIVTDEEMQKKAEEFGVKITVADCKEKVS
ERNGVLVGEVSSAEGGVVKKRRLYASAGNFAIAELINTEMTLTSQGKGSNFIAFGNEFTKQVANKCFKDN
WTKKSNLQDAVKILILCMETVARKTASVSKQFMIVQTASNADVLKVVEKDRNCGSHHHHHH*

pET47b(+)_*m. mazei*_Connectase_V79A_6His

MTLVIAFIGKNGAVMAGDMREITFEGEKPDREKLEKELYSGSIVTDEEMQKKAEEFGVKITVADCKEKVS
ERNGVLVGEASSAEGGVVKKRRLYASAGNFAIAELINTEMTLTSQGKGSNFIAFGNEFTKQVANKCFKDN
WTKKSNLQDAVKILILCMETVARKTASVSKQFMIVQTASNADVLKVVEKDRNCGSHHHHHH*

pET47b(+)_*m. mazei*_Connectase S81A_6His

MTLVIAFIGKNGAVMAGDMREITFEGEKPDREKLEKELYSGSIVTDEEMQKKAEEFGVKITVADCKEKVS
ERNGVLVGEVSAAEGGVVKKRRLYASAGNFAIAELINTEMTLTSQGKGSNFIAFGNEFTKQVANKCFKD
NWTKKSNLQDAVKILILCMETVARKTASVSKQFMIVQTASNADVLKVVEKDRNCGSHHHHHH*

pET47b(+)_*m. mazei*_Connectase S81G_6His

MTLVIAFIGKNGAVMAGDMREITFEGEKPDREKLEKELYSGSIVTDEEMQKKAEEFGVKITVADCKEKVS
ERNGVLVGEVSGAEGGVVKKRRLYASAGNFAIAELINTEMTLTSQGKGSNFIAFGNEFTKQVANKCFKD
NWTKKSNLQDAVKILILCMETVARKTASVSKQFMIVQTASNADVLKVVEKDRNCGSHHHHHH*

pET47b(+)_*m. mazei*_Connectase S81D_6His

MTLVIAFIGKNGAVMAGDMREITFEGEKPDREKLEKELYSGSIVTDEEMQKKAEEFGVKITVADCKEKVS
ERNGVLVGEVSDAEGGVVKKRRLYASAGNFAIAELINTEMTLTSQGKGSNFIAFGNEFTKQVANKCFKD
NWTKKSNLQDAVKILILCMETVARKTASVSKQFMIVQTASNADVLKVVEKDRNCGSHHHHHH*

pET47b(+)_*m. mazei*_Connectase E83A_6His

MTLVIAFIGKNGAVMAGDMREITFEGEKPDREKLEKELYSGSIVTDEEMQKKAEEFGVKITVADCKEKVS
ERNGVLVGEVSSAAGGVVKKRRLYASAGNFAIAELINTEMTLTSQGKGSNFIAFGNEFTKQVANKCFKDN
WTKKSNLQDAVKILILCMETVARKTASVSKQFMIVQTASNADVLKVVEKDRNCGSHHHHHH*

pET47b(+)_*m. mazei*_Connectase_N125S_6His

MTLVIAFIGKNGAVMAGDMREITFEGEKPDREKLEKELYSGSIVTDEEMQKKAEEFGVKITVADCKEKVS
ERNGVLVGEVSSAEGGVVKKRRLYASAGNFAIAELINTEMTLTSQGKGSNFIAFGSEFTKQVANKCFKDN
WTKKSNLQDAVKILILCMETVARKTASVSKQFMIVQTASNADVLKVVEKDRNCGSHHHHHH*

pET47b(+)_*m. mazei*_Connectase_N125G_6His

MTLVIAFIGKNGAVMAGDMREITFEGEKPDREKLEKELYSGSIVTDEEMQKKAEEFGVKITVADCKEKVS
ERNGVLVGEVSSAEGGVVKKRRLYASAGNFAIAELINTEMTLTSQGKGSNFIAFGGEFTKQVANKCFKDN
WTKKSNLQDAVKILILCMETVARKTASVSKQFMIVQTASNADVLKVVEKDRNCGSHHHHHH*

pET47b(+)_*m. mazei*_Connectase_N125A_6His

MTLVIAFIGKNGAVMAGDMREITFEGEKPDREKLEKELYSGSIVTDEEMQKKAEEFGVKITVADCKEKVS
ERNGVLVGEVSSAEGGVVKKRRLYASAGNFAIAELINTEMTLTSQGKGSNFIAFGAEFTKQVANKCFKDN
WTKKSNLQDAVKILILCMETVARKTASVSKQFMIVQTASNADVLKVVEKDRNCGSHHHHHH*

pSNAP_PGA_ (FDADP LVVEI)_SNAP_6His

MPGAFDADPLVVEIGLPVDIKLTGEFAMDKDCEMKRTTLDSPLGKLELSGCEQGLHEIKLLGKGTSAADA
VEVPAPAAVLGGPEPLMQATAWLNAYFHQPEAIEEFPVPALHHPVFQQESFTRQVLWKLLKVVKFGEVI
SYQQLAALAGNPAATAAVKTALSGNPVPILIPCHRVVSSSGAVGGYEGGLAVKEWLLAHEGHRLGKPGL
GPAGGSHHHHHH*

pET47b(+)_6His_3C_Ubquitin_(RELAS) KDPGA (FDADP LVVEI)

MAHHHHHHSAALEVLFQGPGMQIFVKTLTGKTITLEVEPSDTIENVKAKIQDKEGIPPDQQRLIFAGKQL
EDGRTLSDYNIQKESTLHLVLRLRGGNGLRELASKDPGAFDADPLVVEI*

pET47b(+)_PGA (FDADP LVVEI)_Ub_mCherry_6His

MPGAFDADPLVVEIPVDIKMQIFVKTLTGKTITLEVEPSDTIENVKAKIQDKEGIPPDQQRLIFAGKQLEDG
RTLSDYNIQKESTLHLVLRLRLIGEFAGSGSGSVSKGEEDNMAIIKEFMRFKVHMEGSVNGHEFEIEGEGE
GRPYEGTQTAKLKVTKGGPLPFAWDILSPQFMYGSKAYVKHPADIPDYLKLSFPEGFKWERVMNFEDG
GVVTVTQDSSLQDGEFIYKVKLRGTNFPSDGPVMQKKTMGWEASSERMYPEDGALKGEIKQRLKLKDG
GHYDAEVKTTYKAKKPVQLPGAYNVNIKLDITSHNEDYTIVEQYERAEGRHSTGGMDELYKHHHHHH*

pET47b(+)_GGA (VDAKP LVVEI PVDIK)_SNAP_6His

MGGAVDAKPLVVEIPVDIKMDKDCEMKRTTLDSPLGKLELSGCEQGLHEIKLLGKGTSAADAVEVPAPA
AVLGGPEPLMQATAWLNAYFHQPEAIEEFPVPALHHPVFQQESFTRQVLWKLLKVVKFGEVISYQQLAA
LAGNPAATAAVKTALSGNPVPILIPCHRVVSSSGAVGGYEGGLAVKEWLLAHEGHRLGKPGLGHHHHH
H*

pCDNA3.1_EGFP-TM-(5)KDPGA(10)

MVSKGEELFTGVVPILVELDGDVNGHKFSVSGEGEGDATYGKLTLKFICTTGKLPVPWPTLVTTLTYGVQ
CFSRYPDHMKQHDFFKSAMPEGYVQERTIFFKDDGNYKTRAEVKFEGDTLVNRIELKGIDFKEDGNILGH
KLEYNYNSHNVYIMADKQKNGIKVNFKIRHNIEDGSVQLADHYQQNTPIGDGPVLLPDNHYLSTQSALS
KDPNEKRDHMVLLEFVTAAGITLGMDELYKGGGSGSTTSGGSGKKRGNHSTGLCLLVMFFMVLVALVG
LGLGMFQAANAQQETGGGGSGGGSTSTSTTSSAGGGSRELASKDPGAFDADPLVVEISEEGE*

Table S4. Peptides list

Name	Peptide sequence	No. of AA	MW
PGA (P4’)	PGA ADADP LVVEI SEEGE	18	1797
PGA (V P4’)	PGA VDADP LVVEI SEEGE	18	1825
PGA (K P7’)	PGA FDAKP LVVEI SEEGE	18	1887
PGA (P5’)	PGA FAADP LVVEI SEEGE	18	1829
PGA (P8’)	PGA FDADA LVVEI SEEGE	18	1847
PGA (P9’)	PGA FDADP AVVEI SEEGE	18	1831
PGA (P10’)	PGA FDADP LAVEI SEEGE	18	1845
PGA (P11’)	PGA FDADP LVAEI SEEGE	18	1845
PGA (P12’)	PGA FDADP LVVAI SEEGE	18	1815
PGA (P13’)	PGA FDADP LVVEA SEEGE	18	1831
PGA (P14’)	PGA FDADP LVVEI AEEGE	18	1857
5KDPGA10 (P7)	AELAS KDPGA FDADP LVVEI	20	2057
5KDPGA10 (P6)	RALAS KDPGA FDADP LVVEI	20	2084
5KDPGA10 (P5)	REAAS KDPGA FDADP LVVEI	20	2100
5KDPGA10 (P3)	RELAA KDPGA FDADP LVVEI	20	2126
5KDPGA10 (P2)	RELAS ADPGA FDADP LVVEI	20	2085
5KDPGA10 (P1)	RELAS RDPGA FDADP LVVEI	20	2170
AGA 15aa	AGA FDADP LVVEI SEEGE	18	1847
CGA15aa	CGA FDADP LVVEI SEEGE	18	1880
DGA15aa	DGA FDADP LVVEI SEEGE	18	1891
EGA15aa	EGA FDADP LVVEI SEEGE	18	1904
FGA15aa	FGA FDADP LVVEI SEEGE	18	1923
GGA 15aa	GGA FDADP LVVEI SEEGE	18	1832
HGA 15aa	HGA FDADP LVVEI SEEGE	18	1914
IGA 15aa	IGA FDADP LVVEI SEEGE	18	1890
KGA15aa	KGA FDADP LVVEI SEEGE	18	1904
LGA 15aa	LGA FDADP LVVEI SEEGE	18	1890
MGA15aa	MGA FDADP LVVEI SEEGE	18	1908
NGA 15aa	NGA FDADP LVVEI SEEGE	18	1890
PGA 15aa	PGA FDADP LVVEI SEEGE	18	1873
QGA 15aa	QGA FDADP LVVEI SEEGE	18	1905
RGA 15aa	RGA FDADP LVVEI SEEGE	18	1932
SGA 15aa	SGA FDADP LVVEI SEEGE	18	1862
TGA 15aa	TGA FDADP LVVEI SEEGE	18	1877
VGA 15aa	VGA FDADP LVVEI SEEGE	18	1874
WGA15 aa	WGA FDADP LVVEI SEEGE	18	1962
YGA 15aa	YGA FDADP LVVEI SEEGE	18	1939
5KDGGA10	RELAS KDGGA FDADP LVVEI	20	2101
5KDPGA10	RELAS KDPGA FDADP LVVEI	20	2141
5KDPGA10 VK	RELAS KDPGA VDAKP LVVEI	20	2107
